# Identification of Novel Tumor Microenvironment-Related Long Noncoding RNAs to Determine the Prognosis and Response to Immunotherapy of Hepatocellular Carcinoma Patients

**DOI:** 10.3389/fmolb.2021.781307

**Published:** 2021-12-24

**Authors:** Shenglan Huang, Jian Zhang, Xiaolan Lai, Lingling Zhuang, Jianbing Wu

**Affiliations:** ^1^ The Second Affiliated Hospital of Nanchang University, Nanchang, China; ^2^ Jiangxi Key Laboratory of Clinical and Translational Cancer Research, Nanchang, China; ^3^ Ningde Municipal Hospital Affiliated to Ningde Normal University, Ningde, China

**Keywords:** hepatocellular carcinoma, tumor microenvironment, lncRNA, prognostic signature, immune checkpoint inhibitors

## Abstract

**Introduction:** Hepatocellular carcinoma (HCC) is one of the most common malignant tumors with poor prognosis. The tumor microenvironment (TME) plays a vital role in HCC progression. Thus, this research was designed to analyze the correlation between the TME and the prognosis of HCC patients and to construct a TME-related long noncoding RNA (lncRNA) signature to determine HCC patients’ prognosis and response to immunotherapy.

**Methods:** We assessed the stromal–immune–estimate scores within the HCC microenvironment using the ESTIMATE (Estimation of Stromal and Immune Cells in Malignant Tumor Tissues Using Expression Data) algorithm based on The Cancer Genome Atlas database, and their associations with survival and clinicopathological parameters were also analyzed. Thereafter, differentially expressed lncRNAs were filtered out according to the immune and stromal scores. Cox regression analysis was performed to build a TME-related lncRNA risk signature. Kaplan–Meier analysis was used to explore the prognostic value of the risk signature. Furthermore, we explored the biological functions and immune microenvironment features in the high- and low-risk groups. Lastly, we probed the association of the risk model with treatment responses to immune checkpoint inhibitors (ICIs) in HCC.

**Results:** The stromal, immune, and estimate scores were obtained utilizing the ESTIMATE algorithm for patients with HCC. Kaplan–Meier analysis showed that high scores were significantly correlated with better prognosis in HCC patients. Six TME-related lncRNAs were screened to construct the prognostic model. The Kaplan–Meier curves suggested that HCC patients with low risk had better prognosis than those with high risk. Receiver operating characteristic (ROC) curve and Cox regression analyses indicated that the risk model could predict HCC survival exactly and independently. Functional enrichment analysis revealed that some tumor- and immune-related pathways were activated in the high-risk group. We also revealed that some immune cells, which were important in enhancing immune responses toward cancer, were significantly increased in the low-risk group. In addition, there was a close correlation between ICIs and the risk signature, which can be used to predict the treatment responses of HCC patients.

**Conclusion:** We analyzed the influence of the stromal, immune, and estimate scores on the prognosis of HCC patients. A novel TME-related lncRNA risk model was established, which could be effectively applied as an independent prognostic biomarker and predictor of ICIs for HCC patients.

## Introduction

Liver cancer is a common malignant tumor globally (Yoon et al., 2019). More than 800,000 new individuals are diagnosed with liver cancer annually, and the number of liver cancer-related deaths has reached approximately 780,000. Hepatocellular carcinoma (HCC) is the most common liver cancer, accounting for 75%–85% of all primary liver cancers (Siegel et al., 2018). Currently, potentially curative treatments, such as surgical resection, radiofrequency ablation, and liver transplantation, can be applied to patients with early-stage HCC (Demir et al., 2021; Llovet et al., 2021). However, most HCC patients are diagnosed at an advanced stage, when curative treatments are limited, which results in poor prognosis with a 5-year survival rate of no more than 18% (Jemal et al., 2017; Llovet et al., 2021). Recently, immunotherapy for advanced HCC has become a research hotspot, including immune checkpoint blockade (ICB), adoptive cell therapy, oncolytic viruses, and oncolytic bacteria. Among them, ICBs, represented by programmed death-1 (PD-1), programmed death-ligand 1 (PD-L1), and cytotoxic T lymphocyte antigen 4 (CTLA4) blockades, have provided promising treatment options for HCC (Qin et al., 2019). The clinical studies Checkmate-040 (El-Khoueiry et al., 2017) and Keynote-224 (Zhu et al., 2018) have evaluated the efficacy and safety of anti-PD-1 in advanced HCC, and the results demonstrated that the median survival times of patients on nivolumab and pembrolizumab were 15.6 and 12.9 months, respectively. However, PD-1 and PD-L1 inhibitors were only beneficial in ∼20% of HCC patients, and a considerable number of patients did not respond to immunotherapy (Khemlina et al., 2017). Thus, it is of great clinical value to discover novel biomarkers for predicting the survival and clinical response to immunotherapy of HCC patients.

The tumor microenvironment (TME) plays a crucial role in tumorigenesis, progression, immune escape, and therapeutic resistance (Kurebayashi et al., 2018). TME mainly consists of tumor cells, stromal cells, immune cells, and the extracellular matrix (Li et al., 2021). The bidirectional communication between tumor cells and stromal cells or immune cells is critical for maintaining the balance of the TME, which leads to tumor development or suppression (Wu et al., 2021a). Previous studies have demonstrated that monocytes, stimulated by a certain TME, are polarized into the M2 macrophages and secrete anti-inflammatory cytokines such as interleukin-8 (IL-8), interleukin-10 (IL-10), and transforming growth factor beta (TGF-β), which participate in angiogenesis, inflammation, and matrix remodeling, thereby inducing the apoptosis of CD8^+^ T cells, inhibiting T helper 1 (Th1) immune response, and reshaping the TME to promote tumor growth, invasion, and metastasis (Galdiero et al., 2013; Yu et al., 2018; Ovais et al., 2019). In addition, stromal cell types within the TME also play a vital role in cancer development and metastasis. A study found that targeting tumor-associated stromal cells can reduce the risk of treatment resistance and tumor recurrence (Quail and Joyce, 2013). Therefore, a better understanding of the TME of HCC is essential to identifying new prognostic and therapeutic targets, which will consequently predict the response to and efficacy of immunotherapy.

Long noncoding RNAs (lncRNAs), which account for 80%–90% of all ncRNAs, structurally contain more than 200 nucleotides (nt) in length and play indispensable roles in tumorigenesis and progression (Sanchez Calle et al., 2018). Hence, lncRNAs are widely used as tumor biomarkers to determine early diagnosis, prognosis, and potential therapeutic targets (Fan et al., 2016; Necula et al., 2019; Yuan et al., 2020). Moreover, lncRNAs are key players in regulating the TME by mediating the interactions of immune or stromal cells and cancer cells (Wu et al., 2020). Some pieces of evidence suggest that lncRNAs are involved in the process of cancer immunity, including antigen release, antigen presentation, immune cell priming, T-cell activation, and immune cell migration (Wu et al., 2020). It has been reported that the lncRNA UCA1 promoted the proliferation, migration, and immune escape of cancer cells in gastric cancer, induced PD-L1 expression, and decreased the secretion of IFN-γ by restraining the expression levels of miR-26a/b, miR-193a, and miR-214 (Wang et al., 2019). In HCC, lncRNA epidermal growth factor receptor (EGFR) suppressed cytotoxic T lymphocyte (CTL) activity, stimulated regulatory T cell (Treg) differentiation, and accelerated HCC growth *via* blocking EGFR ubiquitination with c-CBL (Jiang et al., 2017). Wang et al. (2020) have revealed that the lncRNA NNT-AS1 impaired CD4^+^ T-cell infiltration *via* activation of the TGF-β signaling pathway in HCC. Moreover, several studies have reported the remarkable role of an lncRNA risk model for survival prediction in HCC (Sun et al., 2019; Xu et al., 2021a; Zhou et al., 2021a), as well as including immune-related lncRNAs, which was capable of predicting the survival and therapeutic responses of HCC patients (Zhang et al., 2020; Wang et al., 2021a; Wu et al., 2021b; Xu et al., 2021b; Zhou et al., 2021b). However, the TME-related lncRNA signature for predicting prognosis requires further exploration. Thus, we focused on the TME of HCC as the cut-in point to discover novel prognostic TME-related lncRNA markers and further investigate latent molecular mechanisms.

**FIGURE 1:**
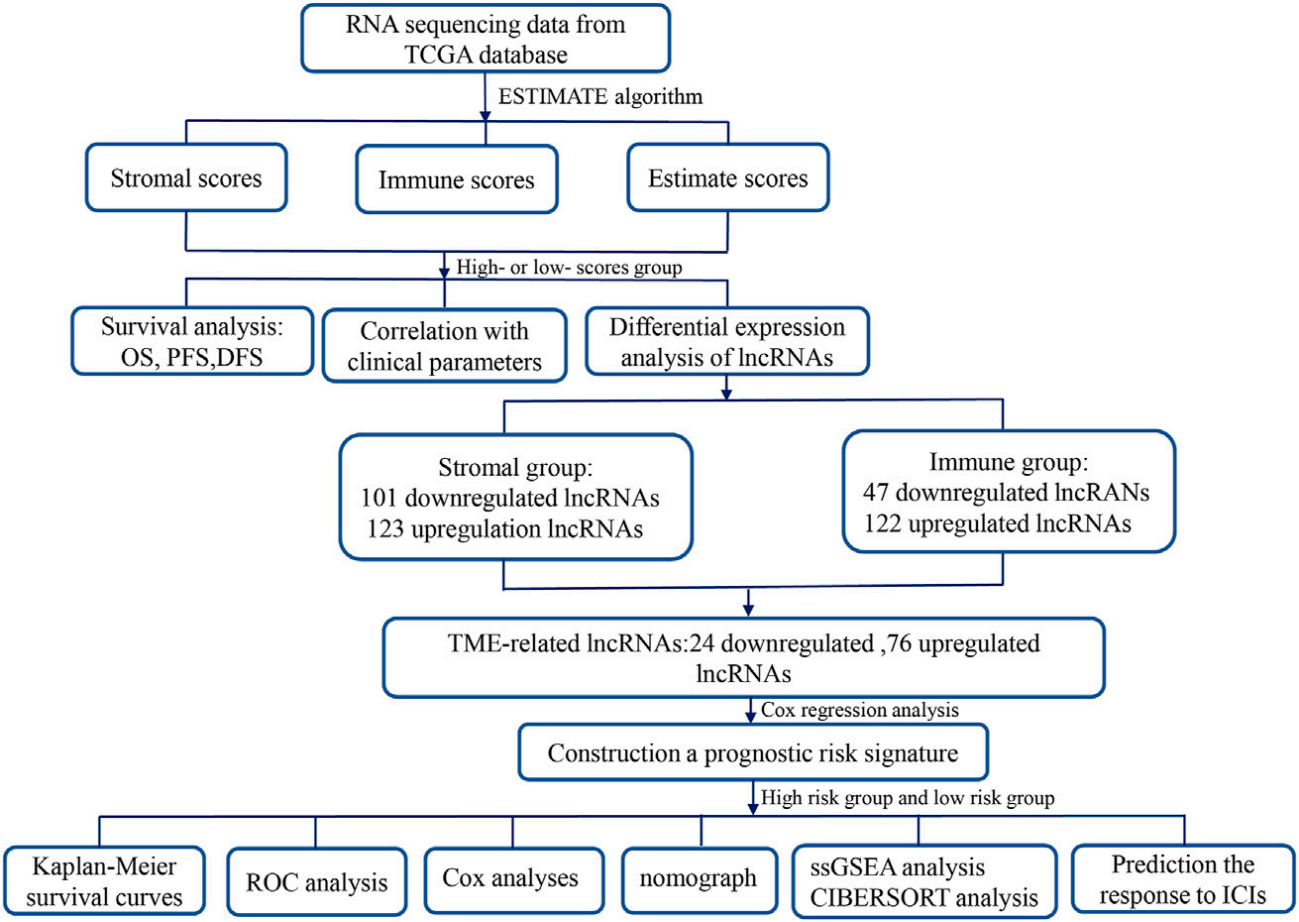
Flowchart of this study. *TME*, tumor microenvironment; *lncRNAs*, long noncoding RNAs; *TCGA*, The Cancer Genome Atlas; *OS*, overall survival; *PFS*, progression-free survival; *DFS*, disease-free survival; *ROC*, receiver operating characteristic; *COX*, univariate and multivariate Cox proportional hazards regression; *GSEA*, gene set enrichment analysis; *GO*, Gene Ontology; *ICIs*, immune checkpoint inhibitors.

This study, we first utilized the ESTIMATE (Estimation of Stromal and Immune Cells in Malignant Tumor Tissues Using Expression Data) algorithm to assess the stromal–immune–estimate scores of the HCC microenvironment based on The Cancer Genome Atlas (TCGA) database and analyzed the associations of these scores with survival and clinicopathological parameters. Subsequently, we constructed a novel TME-related lncRNA risk model, which was filtered out by integrating differentially expressed lncRNAs (DElncRNAs) into the TME, and identified its prognostic value in HCC patients. The latent biological mechanisms were investigated *via* functional enrichment analysis. In addition, the relationship between the TME-related lncRNA signatures and specific TME features was investigated in depth. Furthermore, we explored the associations between the signature and ICB-related genes and applied the TME-related lncRNAs to forecast the treatment response of HCC patients to immune checkpoint inhibitors (ICIs). A flowchart of this research design is shown in Figure 1.

## Materials and Methods

### Data Acquisition and Processing

The RNA sequencing (RNA-seq) data of 374 tumor samples and 50 normal samples were downloaded from TCGA Genomic Data Commons database (https://portal.gdc.cancer.gov/). The genes were annotated and classified as 14,142 lncRNAs and 19,659 protein-coding genes according to the ENSEMBL database (http://asia.ensembl.org/index. html). LncRNAs with average expression value less than “0.1” were discarded; finally, 3,848 lncRNAs were retained for subsequent differential analysis. Corresponding clinical information were downloaded from the UCSC XENA database (https://xenabrowser.net/), which incorporates gender, age, histological grade, clinical stage, TNM stage, survival status, and survival time [including overall survival (OS), progression-free survival (PFS), and disease-free survival (DFS)]. After excluding patients without follow-up data, the remaining 365 HCC samples with both expression and survival data were included in this study.

### Analysis of the Relationship Between Tumor Microenvironment Scores and Prognosis of Hepatocellular Carcinoma Patients

To evaluate the heterogeneity of the TME for each HCC patient, the ESTIMATE algorithm (Yoshihara et al., 2013) was applied to calculate the stromal and immune scores using the “estimate” package in R 4.0.5 software (version 4.0.5; http://www.r-project.org/), which is a specific method that uses the RNA-seq transcriptome profiles of certain genes to quantitatively estimate the scores of stromal and immune cells in the TME. Stromal scores represent the abundance of stromal cells, immune scores display the presence of infiltrating immune cells in the TME, and estimate scores are the sum of the stromal and immune scores. HCC patients were assigned into a low- or a high-stromal/immune/estimate score group according to the best statistical cutoff values, which were calculated using maximally selected rank statistics. Kaplan–Meier survival curves were used to validate the associations between the stromal, immune, and estimate scores and the OS, PFS, and DFS; a *p-*value <0.05 was considered statistically significant. In addition, we also assessed the association of the stromal, immune, and estimate scores in HCC samples with their clinical features, including sex, age, tumor grade, and clinical stage. The results were analyzed and visualized with the “limma” and “ggpubr” packages in R 4.0.5 software; a *p*-value <0.05 represented statistical significance.

### Screening of DElncRNAs Based on the Stromal and Immune Scores

Firstly, 374 HCC samples from TCGA database were divided into high- and low-stromal/immune score subgroups according to the median scores. Thereafter, differential lncRNAs were screened out between the low- and high-score subgroups *via* the “limma” package in R. Wilcoxon’s test was applied to obtain *p*-values, and the significant DElncRNAs were defined as fold change > |±1| and a false discovery rate (FDR) <0.05. The results were visually displayed in volcano plots and heatmaps *via* the packages “ggplot2” and “pheatmap” in R. The heatmaps display the top 20 lncRNAs according to fold change. Subsequently, a Venn diagram was depicted to intersect DElncRNAs that were upregulated or downregulated in both high stromal and immune score subgroups, and the intersecting lncRNAs were defined as TME-related DElncRNAs and used for further analysis.

### Building and Identification of Prognostic Tumor Microenvironment-Related LncRNA Signature for Hepatocellular Carcinoma

To construct a prognostic signature based on the TME-related lncRNAs, we first adopted the univariate Cox regression method to identify lncRNAs related to survival. Thereafter, the multivariate Cox regression method was performed based on the minimum Akaike information criterion (AIC). The prognostic lncRNAs were exhibited with hazard ratio (HR), 95% confidence interval (CI), and *p-*value. A *p* < 0.05 represented the lncRNAs significantly affecting prognosis, lncRNAs with HR > 1 were considered as unfavorable to prognosis, and those with HR < 1 were deemed as improving prognosis. According to the expression value of each lncRNA and its regression coefficient, the risk score of each HCC patient was calculated as follows:

$$\mathrm{Risk}score\;=\;\sum_{i=0}^{n}\exp\times \beta_{i}$$

where exp is the expressive value of each TME-related lncRNA and *β* is the regression coefficient of each lncRNA.

Subsequently, HCC patients were classified into high- and low-risk subgroups based on the median risk score. We applied the Kaplan–Meier curve and log-rank test to compare the survival differences in the two subgroups. The R package “survivalROC” was used to draw the receiver operating characteristic (ROC) curve, and the area under the ROC curve (AUC) was calculated to assess the predictive accuracy of the risk model. Moreover, stratification analysis was performed to evaluate the prognostic value of the risk model in different populations based on the following clinical factors: age (≤65 and >65 years), sex (male and female), grade (grades 1–2 and grades 3–4), and clinical stage (stages I–II and stages III–IV). A *p* < 0.05 was deemed as statistically significant for the above analyses.

Thereafter, a Cox proportional hazards model was derived to identify the independent prognostic factors for HCC patients, including the following clinical features: age, sex, grade, stage, and risk score groups. The results were shown as forest plots, and *p* < 0.05 represented statistical difference.

### Nomogram Building and Validation

According to the risk scores and conventional clinical prognostic indicators (i.e., pathological grade and stage), we built and plotted a nomogram using the R packages “survival” and “rms” to quantitatively calculate the 1-, 2-, and 3-year survival probabilities of HCC patients. Thereafter, the calibration curves and ROC curves were drawn to assess the predictive survival performance of the nomogram.

### Functional Enrichment Analysis

Firstly, gene set enrichment analysis (GSEA) was performed to reveal latent functional enrichment pathways based on the TME-related lncRNA risk signature. We analyzed the gene sets of “c2.cp.kegg.v7.4.symbols.gmt ” obtained from MsigDB (http://www.gseamsigdb.org/gsea/msigdb/collections.jsp) using GSEA 4.1.0, and gene set permutations of 1,000 times were conducted to obtain a standardized enrichment score for each analysis. FDR < 0.05 represented significantly enriched pathways. Subsequently, the significant differentially expressed genes (DEGs) were extracted from TCGA sequencing data depending on risk grouping, and statistical significance was defined as FDR < 0.05 and |log_2_FC| ≥ 1. Based on the DEGs, we conducted Gene Ontology (GO) analysis to discover the related biological pathways in the risk signature.

### Exploring the Correlation of the Tumor Microenvironment–lncRNA Risk Signature With the Tumor Immune Microenvironment

To explore the association of the TME-related lncRNAs with immune cell infiltration in HCC, a range of analytical approaches were conducted. Firstly, single-sample gene set enrichment analysis (ssGSEA) (Rooney et al., 2015) was conducted using the R packages “gsva” and “GSEABase” to explore the differences in the immune cells between the low- and high-risk groups. Thereafter, we obtained the immune cell infiltration information of the HCC samples from the Tumor Immune Estimation Resource (https://cistrome.shinyapps.io/timer/), and CIBERSORT (https://cibersort.stanford.edu/) was used to calculate the relative proportions of 22 tumor-infiltrating immune cells (TIICs) in each HCC patient. Spearman’s correlation analysis was performed to elucidate the correlation of the proportions of TIICs with the risk score. A Spearman’s correlation coefficient (*R*) >1 denotes a positive correlation, and *R* < 1 means a negative correlation; a *p*-value <0.05 was considered statistically significant.

We further investigated the potential values of the risk signature to predict the treatment responses of HCC patients to ICIs. Previous studies have indicated that the immunophenoscore (IPS) is a good predictor of the response to ICIs, and the expressions of immune checkpoint genes are associated with the response to ICIs (Nishino et al., 2017). Firstly, differential expression analysis of the ICI-related genes was performed in the low- and high-risk groups, and we emphatically discussed the correlation of the risk signature with four key genes of ICIs: *CTLA4*, *PD-1*, *PD-L1*, and *PD-L2* (Nishino et al., 2017). Thereafter, the IPS [four subtypes: IPS-CTLA4(−)/PD-1(−), IPS-CTLA4(−)/PD-1(+), IPS-CTLA4(+)/PD-1(−), and IPS-CTLA4(+)/PD-1(+)] was calculated to predict the treatment response to ICIs of the low- and high-risk groups, which were downloaded from the Cancer Immunome Atlas database (https://tcia.at/home). The Wilcoxon rank-sum test was adopted to compare the IPS between the two risk groups, and a *p*-value <0.05 was deemed statistically significant.

### Statistical Analyses

The R 4.0.5 software was used to conduct statistical analyses. Student’s *t*-test or Wilcoxon’s rank-sum test was used to compare the differences between the two groups. Cox regression analysis was performed to build a prognostic model. We applied the Kaplan–Meier method and ROC curve to analyze the survival difference and the predictive ability of the risk signature. Spearman’s correlation analysis was performed to reflect the relationship between the proportions of tumor-infiltrating immune cells and risk scores. All statistical tests were two-sided, and statistical significance was set at *p* < 0.05.

## Results

### The Stromal–Immune–Estimate Scores of the Tumor Microenvironment Were Significantly Associated With the Prognosis of Hepatocellular Carcinoma Patients

**FIGURE 2:**
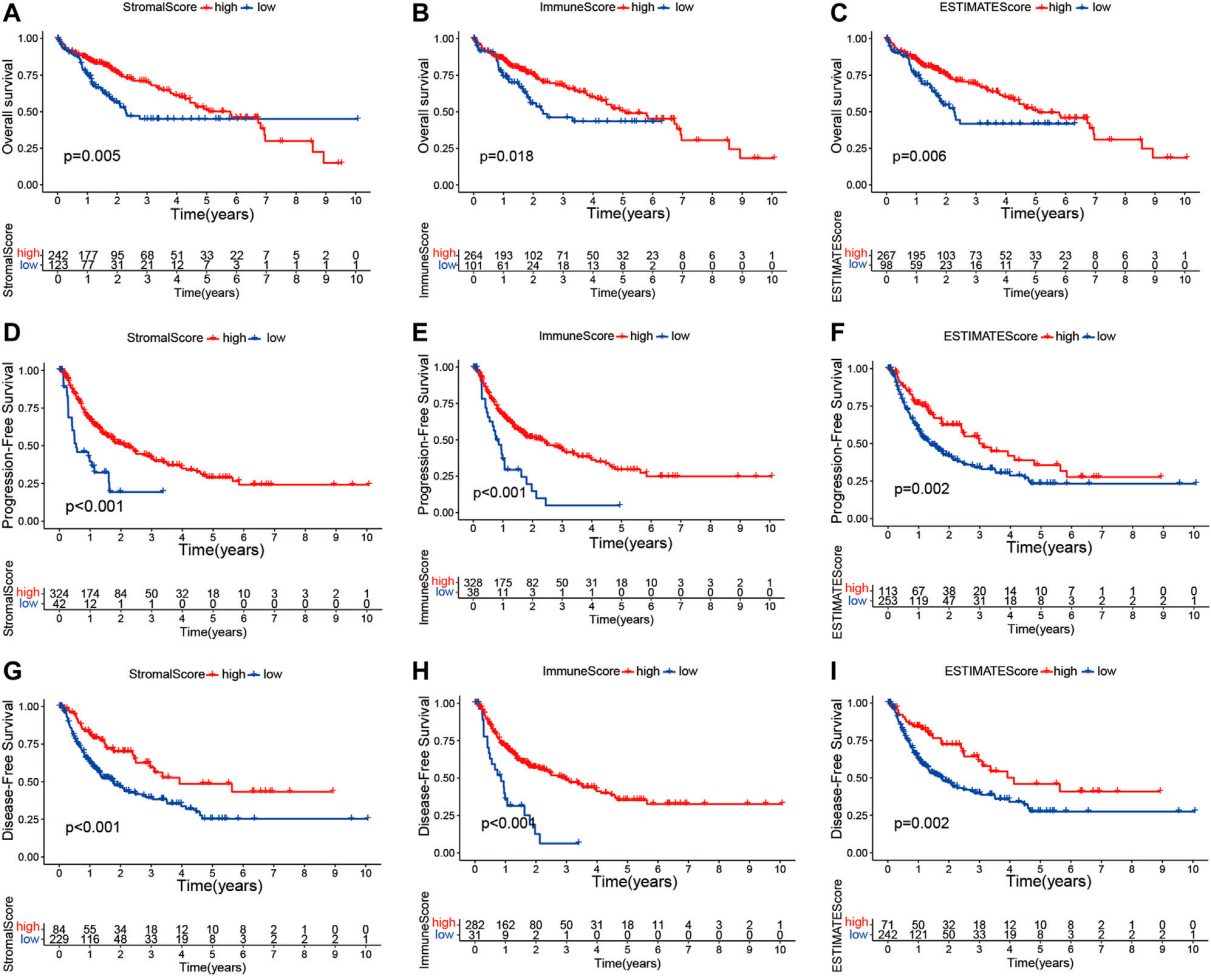
Kaplan–Meier survival curve analysis of the stromal, immune, and estimate scores with the prognosis of HCC patients. **(A–I)** Correlations of the stromal, immune, and estimate scores with overall survival **(A–C)**, progression-free survival **(D–F)**, and disease-free survival **(G–I)**. *HCC*, hepatocellular carcinoma.

The transcriptome and relevant clinical data of HCC patients were downloaded from TCGA and UCSC Xena databases, respectively. The transcriptome data contained 374 tumor and 50 normal tissues, and 377 HCC patients had clinical information. After removing repeated samples and patients without follow-up data, a total of 365 patients were included in the subsequent survival analysis. Thereafter, the stromal, immune, and estimate scores were calculated with the ESTIMATE algorithm (Supplementary Table S1). The 365 HCC patients were split into low- and high-score subgroups in line with the best statistical cutoff values, which were −914.61 (ranging from −1,622.33 to 1,180.26) in the stromal groups, 41.48 (ranging from −861.77 to 3,157.28) in the immune groups, and −909.71 (ranging from −2,165.59 to 3,722.93) in the estimate groups. Subsequently, the Kaplan–Meier survival curves were used to analyze the association of the stromal, immune, and estimate scores with OS (Figures 2A–C), PFS (Figures 2D–F), and DFS (Figures 2G–I). The results revealed that HCC patients with high stromal scores showed longer OS (*p* = 0.005), PFS (*p* < 0.001), and DFS (*p* < 0.001) than did patients with low stromal scores (Figures 2A, D, G). Significantly better OS (*p* = 0.018), PFS (*p* < 0.001), and DFS (*p* < 0.001) were seen in the high-immune score subgroup compared to the low-immune score subgroup. Similar survival results were observed in the estimate score groups. These results suggest that TME scores are remarkably associated with the prognosis of HCC patients and deserve further investigation.

**FIGURE 3:**
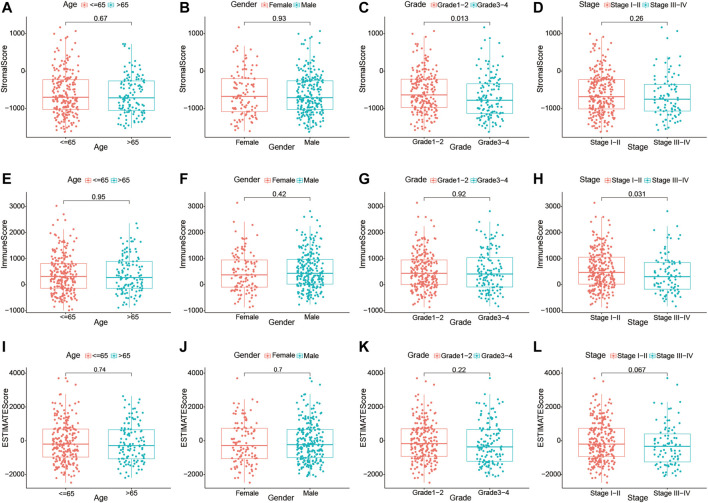
Relationship of the immune, stromal, and estimate scores with the different clinicopathological parameters of hepatocellular carcinoma **(**HCC) patients. **(A–D)** Association of the stromal scores with different age subgroups (≤65 and >65 years), gender subgroups (female and male), grade subgroups (grades I–II and grades III–IV), and stage subgroups (stages I–II and stages III–IV). **(E–H)** Association of the immune scores with different age subgroups (≤65 and >65 years), gender subgroups (female and male), grade subgroups (grades I–II and grades III–IV), and stage subgroups (stages I–II and stages III–IV). **(I–L)** Association of the estimate scores with different age subgroups (≤65 and >65 years), gender subgroups (female and male), grade subgroups (grades I–II and grades III–IV), and stage subgroups (stages I–II and stages III–IV).

Furthermore, we investigated whether there was any association between the stromal, immune, and estimate scores and the clinicopathological characteristics of HCC patients. As shown in Figures 3A–L, no significant association was observed between the estimate scores and gender (male *vs*. female), age (≥65 *vs*. <65 years), tumor grade (grades 1–2 *vs*. grades 3–4), and clinical stage (stages I–II *vs*. stages III–IV). Moreover, the immune and stromal scores showed no significant differences in the age and sex subgroups. However, we noticed that patients in the late stages (stages III–IV) had lower immune scores than those in stages I–II (*p* = 0.031), and a higher grade (grades 3–4) was associated with lower stromal scores (*p* = 0.013). These results demonstrate that the presence of immune and stromal cells in the TME might play an important role in HCC progression.

### Identification of DElncRNAs Based on Low and High Stromal and Immune Scores

**FIGURE 4:**
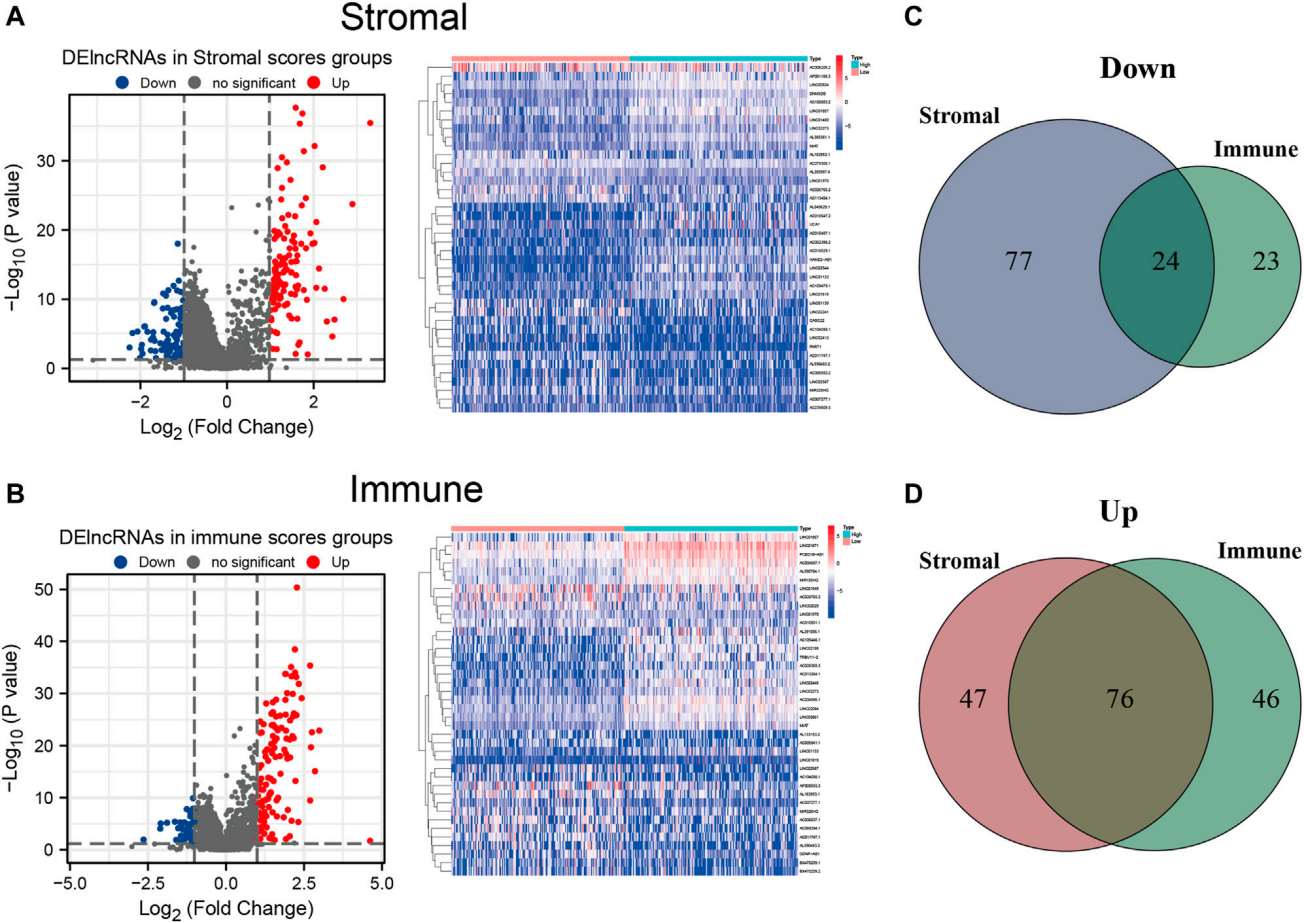
Differentially expressed long noncoding RNAs (DElncRNAs) between the high- and low-stromal/immune score groups. **(A**,**B)** Volcano plot and heatmap of the DElncRNAs in the high- and low-stromal score groups **(A)** and in the high- and low-immune score groups **(B)**. **(C)** Downregulated lncRNAs both in the high-stromal score and high-immune score groups using a Venn diagram. **(D)** Upregulated lncRNAs both in the stromal score and immune score groups using a Venn diagram.

To establish a plausible connection between lncRNAs and the TME, hoping to discover novel prognostic predictors for HCC patients, we conducted lncRNA differential expression analysis of the 374 HCC samples from TCGA cohort. Firstly, the patients were partitioned into a low- or a high-score group based on the median values of the stromal and immune scores. DElncRNAs were filtered out between the low- and high-stromal/immune score groups. The results were visualized with volcano plots and heatmap. Moreover, 101 downregulated and 123 upregulated lncRNAs were identified in the high-stromal score group (Figure 4A and Supplementary Table S2), and 47 downregulated and 122 upregulated lncRNAs were identified in the high-immune score group (Figure 4B and Supplementary Table S3). Among them, the intersecting lncRNAs that were commonly downregulated or upregulated both in the high-stromal and high-immune score subgroups were considered most likely associated with the TME of HCC. Thus, compared with the low-score groups, 24 downregulated and 76 upregulated lncRNAs were selected as TME-related DElncRNAs, as shown in the Venn diagram (Figures 4C, D and Supplementary Table S4).

### Construction of a Prognostic Risk Signature Using the Tumor Microenvironment-Related DElncRNAs and Exploring Its Prognostic Value

Cox regression analyses were conducted to explore the association between the TME-related DElncRNAs and the prognosis of HCC patients. Firstly, among the 100 TME-related DElncRNAs, 9 lncRNAs were screened out as prognosis-related candidates using univariate Cox regression analysis (*p* < 0.05). These lncRNAs are listed in Table 1. Subsequently, multivariate Cox regression analysis was performed to identify the prognostic lncRNA signature with a minimum AIC value (AIC = 1,254), which contained six TME-related lncRNAs (LINC01150, LINC02273, LINC00426, AP002954.1, AC007277.1, and AC008549.1). Among them, LINC02273, AC008549.1, and LINC00426 benefited prognosis, with a HR < 1, whereas LINC01150, AP002954.1, and AC007277.1 were poor prognostic factors with HR > 1 (Table 2).

**TABLE 1:** Univariate Cox regression analysis of TME-related lncRNAs

LncRNA	HR	HR.95L	HR.95H	*p*-value
LINC01150	2.279	1.421	3.655	0.0006
LINC02273	0.245	0.062	0.973	0.046
LINC00426	0.187	0.037	0.938	0.042
LINC01094	2.007	1.393	2.891	0.0002
LINC00892	0.246	0.061	0.989	0.048
LINC01871	0.907	0.823	0.999	0.049
AP002954.1	1.583	1.000	2.504	0.05
AC007277.1	1.242	1.009	1.527	0.04
AC008549.1	0.985	0.972	0.998	0.025

*TME*, tumor microenvironment; *lncRNA*, long noncoding RNA; *HR*, hazard ratio; *HR.95L*, low 95% confidence interval (CI) of HR; *HR.95H*, high 95%CI of HR

**TABLE 2:** Multivariate Cox regression analysis of TME-related lncRNAs

LncRNA	Coefficient	HR	HR.95L	HR.95H	*p*-value
LINC01150	0.947	2.579	1.524	4.365	0.0004
LINC02273	−1.838	0.159	0.024	1.036	0.054
LINC00426	−3.083	0.046	0.005	0.451	0.008
AP002954.1	1.255	3.507	1.71	7.192	0.0006
AC007277.1	0.179	1.195	0.997	1.433	0.054
AC008549.1	-0.014	0.986	0.974	0.999	0.039

*TME*, tumor microenvironment; *lncRNA*, long noncoding RNA; *HR*, hazard ratio; *HR.95L*, low 95% confidence interval (CI) of HR; *HR.95H*, high 95% CI of HR

**FIGURE 5:**
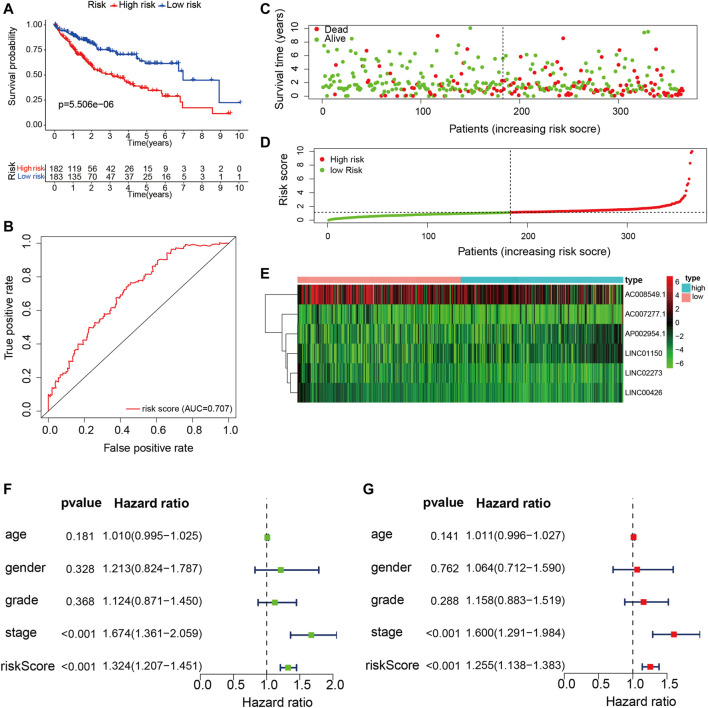
Construction and validation of the tumor microenvironment (TME)-related long noncoding RNA (lncRNA) signature in hepatocellular carcinoma (HCC) samples. **(A)** Kaplan–Meier survival analysis of the high- and low-risk groups. **(B)** Receiver operating characteristic (ROC) curve evaluating the predictive accuracy of the prognostic model. **(C)** Risk score curves based on the risk score of each HCC patient. **(D)** Scatter plots displaying the survival status of each HCC patient. **(E)** Heatmap exhibiting the expression levels of the six TME-related lncRNAs in the high- and low-risk groups. **(F)** Univariate Cox regression analysis of the clinical features and risk scores in HCC samples. **(G)** Multivariate Cox regression analysis of clinical features and risk scores in HCC samples.

According to the expression values of the six TME-related lncRNAs and the multivariate Cox regression coefficients, the risk score of each patient was calculated basing on the following equation: risk score = (0.947 × LINC01150 expression) + (−0.838 × LINC02273 expression) + (−3.083 × LINC00426 expression) + (1.255 × AP002954.1 expression) + (0.178 × AC007277.1 expression) + (−0.014 × AC008549.1 expression). The 365 HCC patients were divided into two groups according to the median score (median value = 1.119). Kaplan–Meier analysis suggested that HCC patients with low risk had longer OS than those with high risk (*p* = 5.506E−06). The results are shown in Figure 5A. The ROC curve demonstrated that the TME-related signature was effective in predicting the OS of HCC patients, with an AUC of 0.707 (Figure 5B). The risk score curve and the scatter plot revealed that the risk score was proportional to the number of deaths in HCC patients (Figures 5C, D). The heatmap indicted that the expressions of LINC01150, AP002954.1, and AC007277.1 were upregulated in the high-risk group, whereas the other three lncRNAs (LINC02273, LINC00426, and AC008549) were upregulated in the low-risk group (Figure 5E). Furthermore, univariate and multivariate Cox proportional hazards regression analyses were performed to explore the independent prognostic factors for OS. The results suggested that the clinical stage and risk score were independent predictors (Figures 5F, G). The above results demonstrated that the TME-related lncRNA risk model could act as a prospective independent predictor for the survival of HCC patients.

**FIGURE 6:**
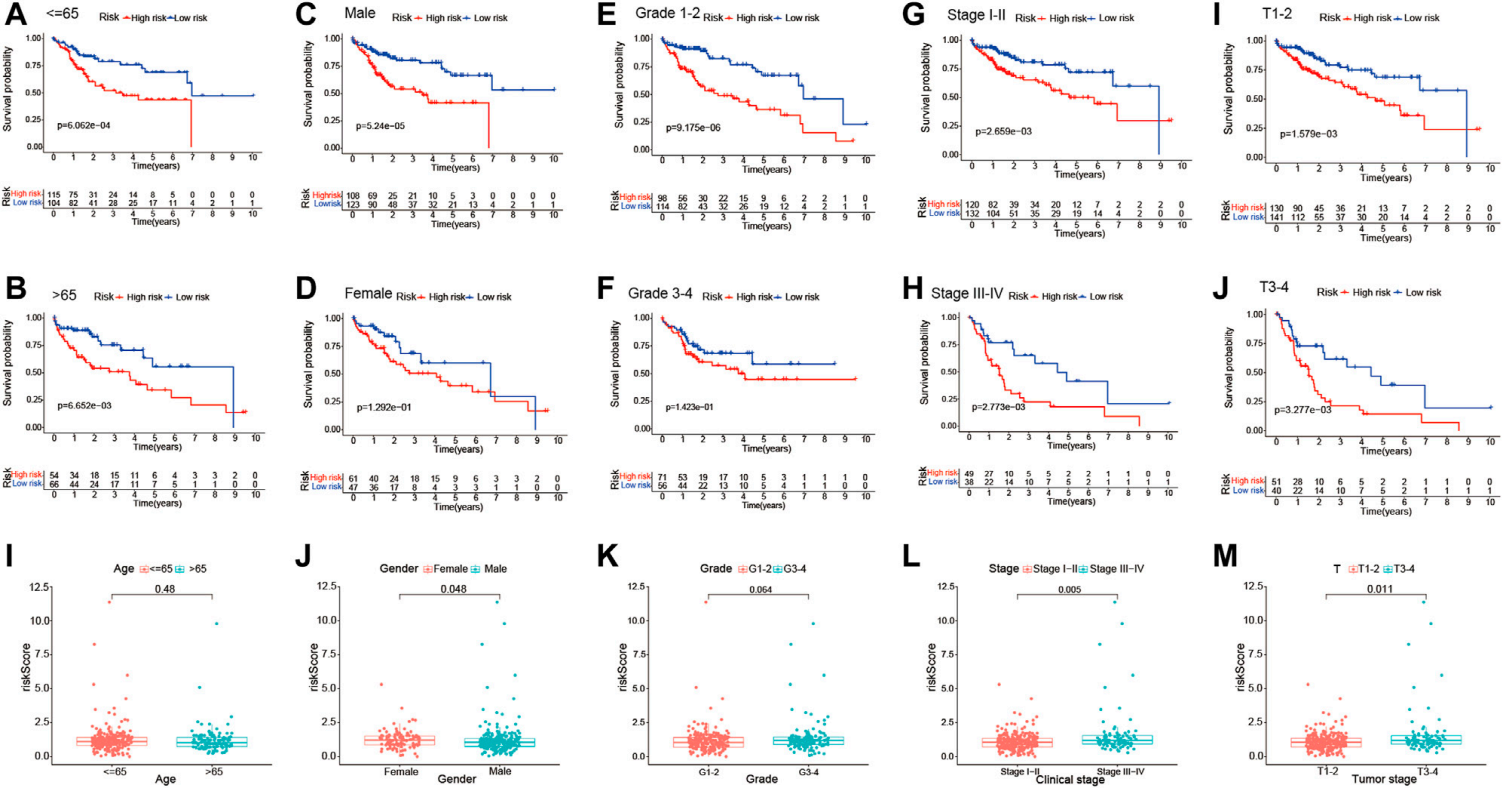
Subgroup analysis of the correlation of clinical features with the risk signature. **(A–J)** Kaplan–Meier survival curve analysis of the risk group with age subgroups (≤65 and >65 years), gender subgroups (female and male), grade subgroups (grades I–II and grades III–IV), and stage subgroups (stages I–II and stages III–IV), and tumor stage subgroups (T1–T2 and T3–T4). **(I–M)** Differential analysis of the risk scores in different subgroups using the Student’s *t*-test or Wilcoxon’s rank-sum test.

In addition, stratification analysis was conducted in different subgroups to investigate the prognostic value of the risk signature. Firstly, the HCC patients were categorized into different subgroups based on age (≤65 and >65 years), sex (male and female), grade (grades 1–2 and grades 3–4), clinical stage (stages I–II and stages III–IV), and tumor stage (T1–T2 and T3–T4); then, Kaplan–Meier survival curve analysis was performed in different subgroups. As shown in Figures 6A–H, patients of different ages and in different tumor and clinical stages in the low-risk group had longer OS than those in the high-risk group; similar results were obtained in the male and grade 1–2 subgroups. However, there were no significant differences in female and grade 3–4 patients. The association between the risk scores of the TME-related lncRNAs and the clinical features of HCC was assessed, and it was found that the prognostic model was significantly correlated with gender (*p* = 0.048), tumor stage (*p* < 0.001), and clinical stage (*p* = 0.011), but not with age or tumor grade (Figures 6I–N). Collectively, it can be concluded that the risk signature could predict the survival of diverse populations of HCC patients, and the risk score may be associated with HCC progression.

### The Nomogram Exact Prediction of the Survival of Hepatocellular Carcinoma Patients

**FIGURE 7:**
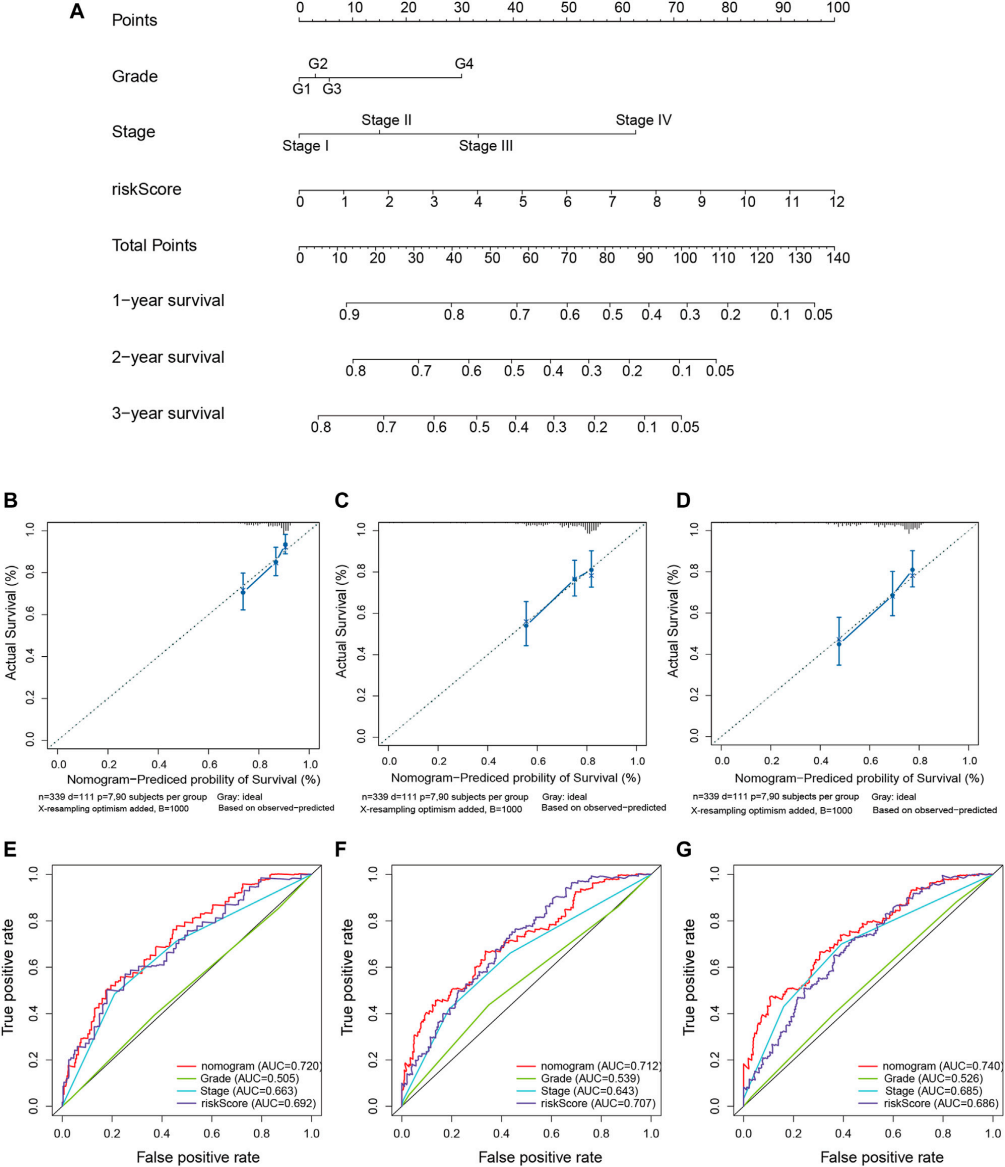
Construction of the predictive nomogram for hepatocellular carcinoma (HCC) patients from The Cancer Genome Atlas (TCGA). **(A)** Nomogram predicting the probability of the 1-, 2-, and 3-year overall survival (OS) of HCC patients. **(B–D)** Calibration curve showing the predictive accuracy of the nomogram. The *Y*-axis represents actual survival, whereas the *X*-axis represents the nomogram-predicted survival. **(E–G)** Receiver operating characteristic (ROC) curve analysis assessing the predictive performance of the nomogram for 1-, 2-, and 3-year OS.

We quantitatively calculated the survival probabilities of HCC patients according to the pathological grade, stage, and risk score by constructing a nomogram. The points for each clinical factor were summed to comprehensive scores. The nomogram showed higher total points and worse prognoses (Figure 7A). The consistency and accuracy of the nomogram were evaluated using a calibration curve and the ROC curves. The calibration curves showed that the nomogram could precisely predict the probability of OS (Figures 7B–D), and ROC analysis indicated that the nomogram achieved better prediction performance than did the other clinical features, with AUCs of 0.720 for 1-year OS, 0.712 for 2-year OS, and 0.740 for 3-year OS, as shown in Figures 7E–G. In summary, the nomogram exhibited preferable clinical practicality for predicting the survival probability of HCC patients.

### Potential Biological Pathway and Functional Enrichment Analysis Based on the Risk Signature

**FIGURE 8:**
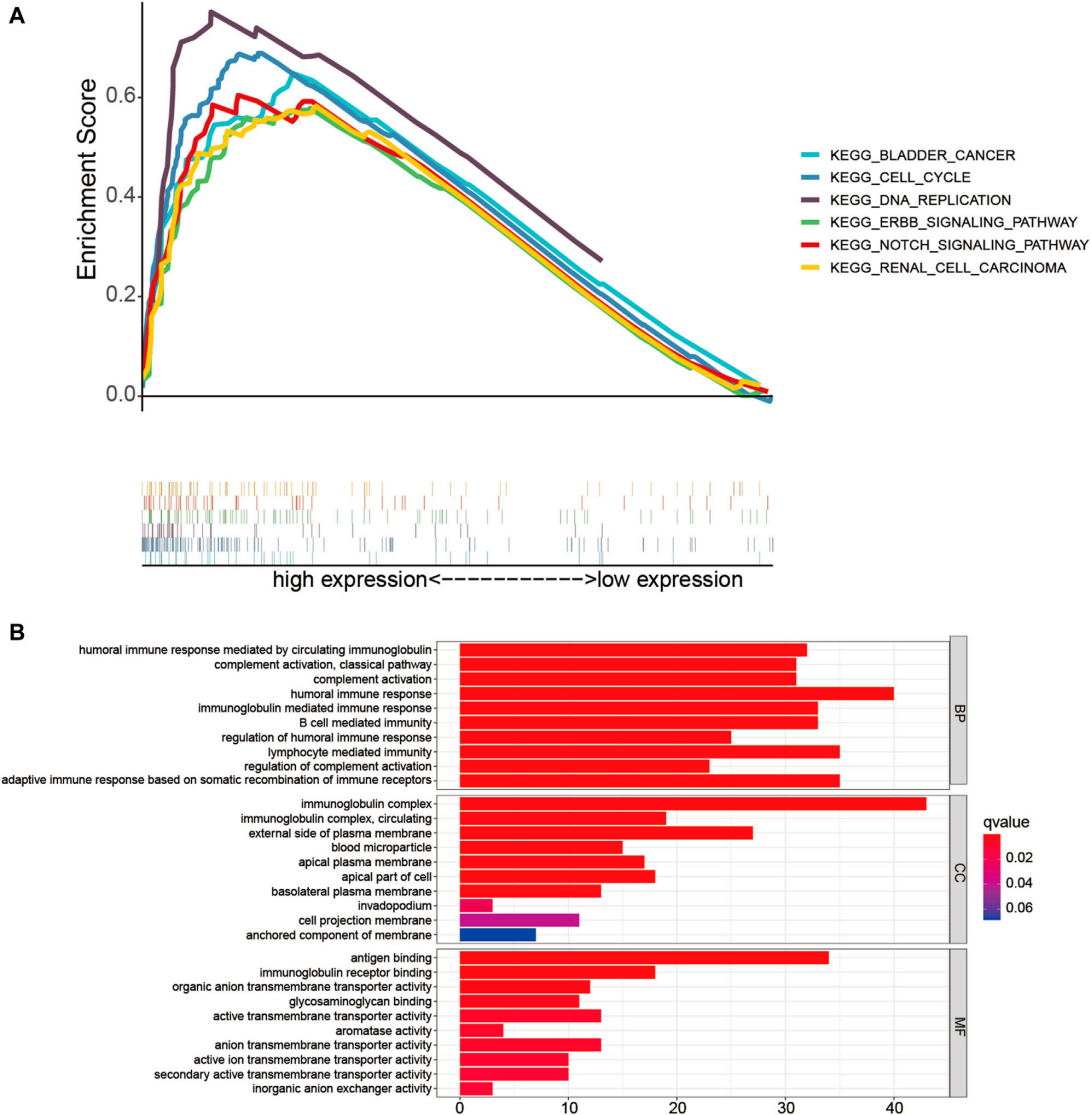
Functional enrichment analysis of the low- and high-risk groups. **(A)** Gene set enrichment analysis (GSEA) based on risk grouping in the cohort from The Cancer Genome Atlas (TCGA). **(B)** Gene Ontology (GO) analysis of the differentially expressed genes (DEGs) based on the high- and low-risk groups.

Firstly, GSEA was performed to probe the latent functional enrichment pathways of the TME-related lncRNAs signature, and the results revealed that tumor-related pathways, including bladder cancer, renal cell carcinoma, DNA replication, cell cycle, ErbB, and Notch signaling pathway, were highly enriched in the high-risk group (*p* < 0.05; Figure 8A). To further determine the possible biological pathways associated with the risk signature, DEGs were selected from the high- and low-risk groups based on the criteria |log2FC| ≥ 1 and *p* < 0.05. Compared with the low-risk group, a total of 256 DEGs (119 upregulated genes and 136 downregulated genes) were identified in the high-risk group. We then selected DEGs for GO enrichment analysis. The results indicated that certain pathways related to immune response were enriched, such as “humoral immune response,” “complement activation,” “adaptive immune response,” and “B-cell-mediated immunity” (Figure 8B). Thus, we can conclude that the prognostic TME-related lncRNA signature might play an important role in reshaping the tumor immune microenvironment, tumorigenesis, and progression of HCC.

### The Prognostic Risk Signature Affected Tumor Immune Features and Predicted Treatment Response to Immune Checkpoint Inhibitors

**FIGURE 9:**
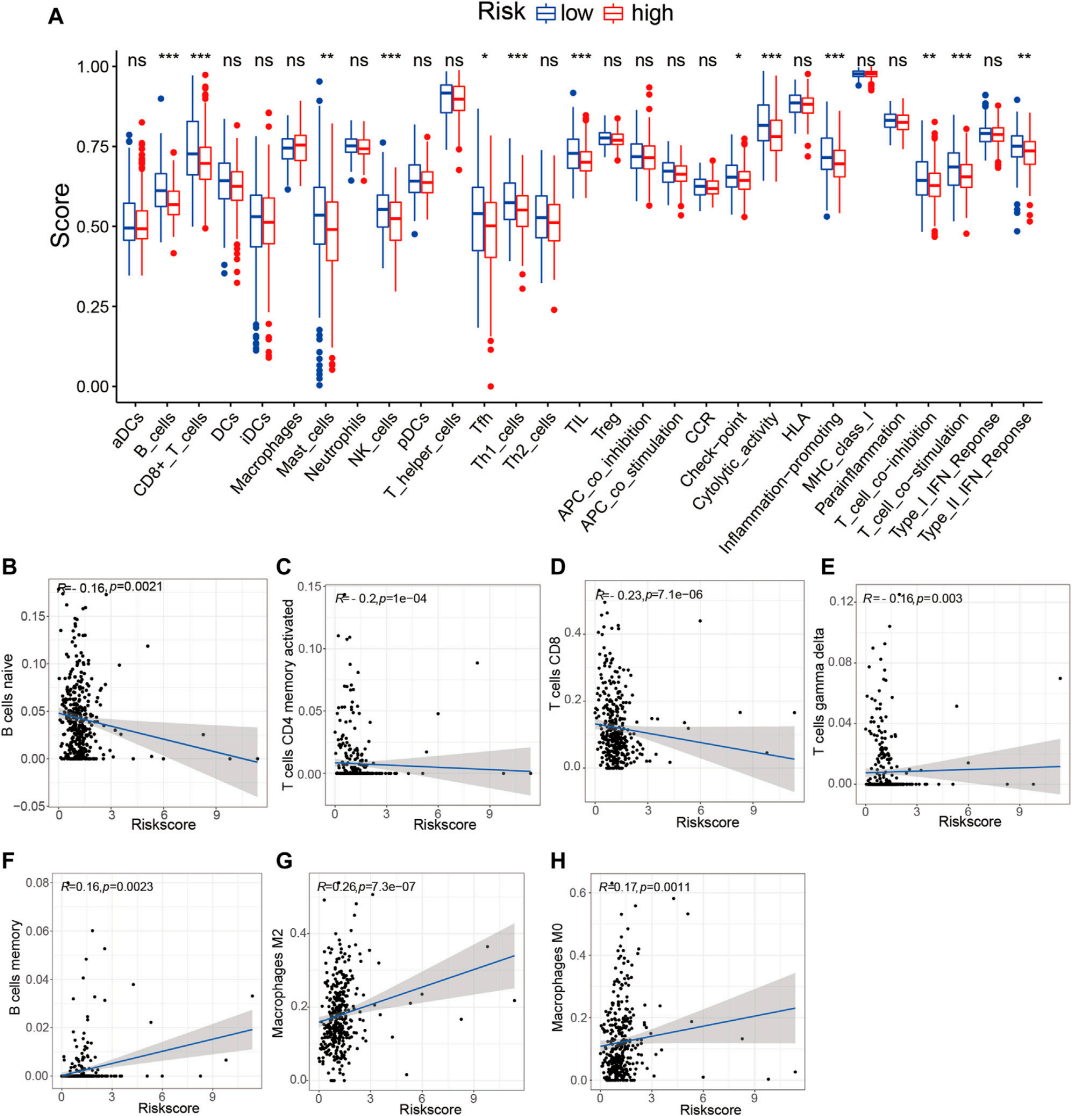
Tumor immune microenvironment state analysis of the tumor microenvironment (TME)-related long noncoding RNA (lncRNA) signature using ssGSEA and the CIBERSORT algorithm. **(A)** Comparison of the immune cell infiltration and immune functions between the low- and high-risk groups. **(B–H)** Correlation analysis between the immune cell infiltration levels and risk scores. *p < 0.05; **p < 0.01; ***p < 0.001. *ssGSEA*, single-sample gene set enrichment analysis.

To determine whether the TME-related lncRNA signature influences the infiltration levels of immune cells in HCC, we evaluated the differences in the immune cells between the high- and low-risk groups in HCC patients using ssGSEA and the CIBERSORT algorithm. From the ssGSEA results, we found that the immune cells, including B cells, CD8^+^ T cells, mast cells, follicular Th cells, Th1 cells, and tumor-infiltrating lymphocytes (TILs), were obviously increased in the low-risk set, and some pathways related to immune function (i.e., immune checkpoint pathway, cytolytic activity, inflammation-promoting, T-cell co-inhibition/stimulation, and INF-II response) were significantly activated in the low-risk group compared with that in the high-risk group (*p* < 0.05; Figure 9A). Moreover, the CIBERSORT analysis results suggested that the following immune cell infiltration levels were negatively associated with the risk score: naive B cells (*R* = −0.16, *p* = 0.0021), activated memory CD4^+^ T cells (*R* = −0.2, *p* = 1e−04), CD8^+^ T cells (*R* = −0.23, *p* = 7.1e−06), and γδT cells (*R* = −0.16, *p* = 0.003). On the other hand, a significant positive correlation was observed in the proportions of memory B cells (*R* = 0.16, *p* = 0.0021), M0 macrophages (*R* = 0.26, *p* = 7.3e−07), and M2 macrophages (*R* = 0.17, *p* = 0.0011) with the risk score (Table 3 and Figures 9B–H). These findings indicate that the signature of six TME-related lncRNAs affected the immune cell infiltration in HCC.

**TABLE 3:** Correlation analysis of the 22 immune cell infiltration levels with the risk scores

Immune cells	*R*	*p*-value
Naive B cells	−0.16	0.002095003
Memory B cells	0.16	0.002336936
Plasma cells	−0.1	0.05199941
CD8^+^ T cells	−0.23	7.15E−06
Naive CD4^+^ T cells	−0.059	0.263867315
Resting memory CD4 T cells	0.013	0.810632983
Activated memory CD4 T cells	−0.2	0.000102883
Follicular helper T cells	−0.013	0.807812793
Regulatory T cells (Tregs)	−0.055	0.30002029
Gamma delta T cells	−0.16	0.002975049
Resting NK cells	−0.088	0.095016394
Activated NK cells	0.0096	0.855642376
Monocytes	0.061	0.250214059
Macrophages M0	0.17	0.001087181
Macrophages M1	−0.065	0.216904497
Macrophages M2	0.26	7.33E−07
Resting dendritic cells	−0.017	0.743538196
Activated dendritic cells	0.1	0.051715294
Resting mast cells	−0.023	0.666050316
Activated mast cells	0.00092	0.986067845
Eosinophils	−0.037	0.4874298
Neutrophils	0.089	0.09158816

*R*, Spearman’s rank correlation rho; *NK cells*, natural killer cells.

**FIGURE 10:**
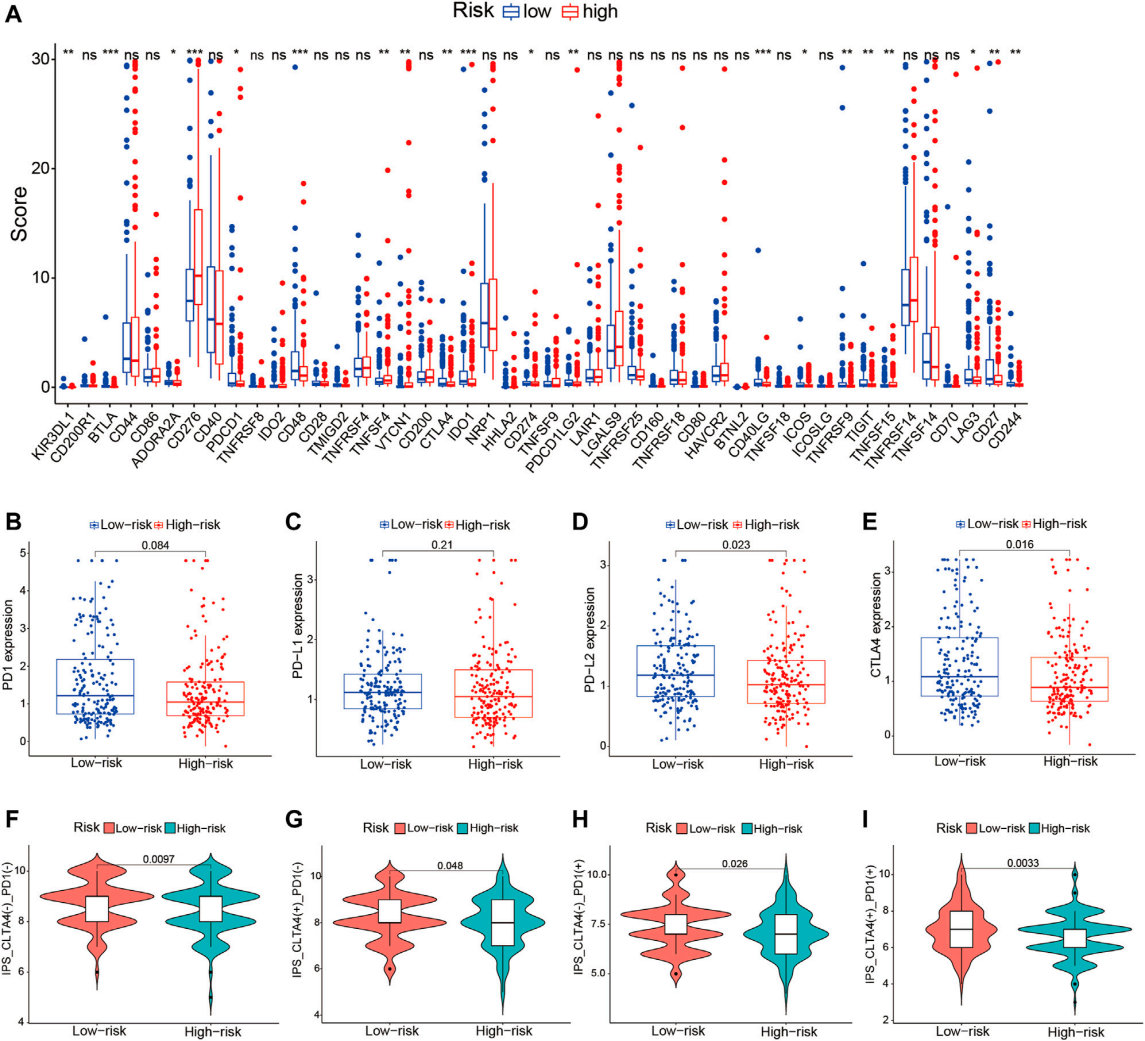
Correlation of the tumor microenvironment (TME)-related long noncoding RNA (lncRNA) signature with ICI-related genes and treatment response to ICIs. **(A)** Comparison of the expression levels of 46 ICI-related genes in the high- and low-risk score groups. **(B–E)** Differences in the expression levels of *PD-1*, *PD-L1*, *PD-L2*, and *CTLA4* between the high- and low-risk groups. **(F–I)** Comparison of the treatment response to ICIs in the two risk score groups. *ICIs*, immune checkpoint inhibitors.

Given the significant differences in the immune checkpoint pathways in the two risk groups, we further investigated whether the risk signature could predict the treatment response to ICIs. We first explored the relationship between the TME-related lncRNAs signature and 46 ICI-related genes and concentrated on the four pivotal ICI-related genes (*PD-1*, *PD-L1*, *PD-L2*, and *CTLA4*). We found significant expression differences in 20 ICI-related genes between the high- and low-risk groups (*p* < 0.05; Figure 10A). Among the four key genes, the expression levels of *PD-L2* and *CTLA4* were significantly upregulated in the low-risk group (Figures 10B–E), indicating that the risk signature might play a vital role in predicting the treatment response of HCC patients to ICIs. Thus, we further verified the association of the risk signature with the IPS in HCC samples. The results showed that patients in the low-risk group exhibited higher IPS, involving four IPS subtypes [IPS-CTLA4(−)/PD-1(+), IPS-CTLA4(−)/PD-1(+), IPS-CTLA4(+)/PD-1(−), and IPS-CTLA4(+)/PD-1(+)], suggesting that HCC patients with low risk might benefit more from ICI therapy (Figures 10F–I).

## Discussion

HCC is one of the most prevalent malignancies with high mortality rates, high recurrence rates, and poor prognosis. Increasing evidence suggests that the TME plays a vital role in tumorigenesis and progression (Li et al., 2021). Thus, we focused on the TME of HCC as an entry point to discovering novel prognostic markers. In this study, we found that the stromal, immune, and estimate scores of the TME were significantly positively correlated with longer survival, including OS, PFS, and DFS, in patients with HCC. The TME-related lncRNA risk signature was directly constructed from the DElncRNAs of TME *via* Cox regression analysis. The Kaplan–Meier survival curve analysis manifested that the survival in the high-risk group was worse than that in the low-risk group, and the risk signature could accurately and independently predict the prognosis of HCC patients. In addition, we discovered that the TME-related lncRNA risk signature could significantly affect the infiltration levels of immune cells, and the prognostic risk signature might be able to predict the clinical response of HCC patients to ICIs.

HCC is an inflammation-associated malignancy that comprise numerous immune cell subtypes (i.e., tumor-associated macrophages, tumor-associated neutrophils, and myeloid-derived suppressor cells), forming a complex immune tolerance microenvironment and contributing to the development of HCC (Nishida and Kudo, 2017; Kurebayashi et al., 2018). In recent years, with the wide application of bioinformatics, increasing numbers of research works have applied statistical algorithms to investigate the characteristics of the TME and explore new targets for immunotherapy (Xiang et al., 2021). The study by Li et al. reported that the TME of HCC could be classified into different molecular subtypes based on the immune gene expression profiles, which exhibited different immunogenetic features and survival outcomes (Li et al., 2019a). In this study, the ESTIMATE algorithm was used to explore the correlation of the TME scores (including the stromal, immune, and estimate scores) with survival and clinicopathological parameters in HCC. The results showed that higher TME scores were significantly correlated with longer survival, including OS, PFS, and DFS. Moreover, the immune and stromal scores were inversely correlated with tumor grade and clinical stage. Our results are in accordance with a previous study reporting that the stromal and immune scores were correlated with the 3-year OS of HCC patients (Tian et al., 2020). Therefore, the stromal, immune, and estimate scores might predict the survival of HCC patients and the malignancy of tumors.

According to previous studies, lncRNAs were reported to be indispensable regulators of the TME, ultimately affecting tumor behaviors (Wu et al., 2020; Atianand et al., 2017). For instance, lncRNA-ROR, a stress-responsive lncRNA highly expressed in HCC cells and enriched in extracellular vesicles derived from tumor cells, could induce primary chemoresistance by activating the TGF-β pathway in HCC (Takahashi et al., 2014). Moreover, immune-related lncRNA signatures have been reported as prognostic biomarkers and therapeutic targets in several tumors, including lung adenocarcinoma, bladder cancer, esophageal cancer, gastric cancer, rectal cancer, cervical cancer, and HCC (Chen et al., 2021; Lai et al., 2021; Liu et al., 2021; Nie et al., 2021; Pang et al., 2021; Song et al., 2021; Xing et al., 2021; Ye et al., 2021), indicating that immune-related lncRNAs could effectively be applied to determine the survival, prognosis, and treatment response. However, all these studies focused on excerpting the immune-related lncRNA profiles from the obtained immune-related genes based on co-expression analysis while ignoring the important role of the stromal components in tumor progression; immune-related genes remain inconclusive in different research studies, and there are no uniform criteria for filtering immune-related lncRNAs, which may limit the generalization of the predictive model. Hence, we directly screened DElncRNAs based on the stromal and immune scores to construct a novel prognostic TME-related lncRNA marker, which were considered most likely associated with the TME of HCC. Eventually, we identified six TME-related lncRNAs (LINC01150, LINC02273, LINC00426, AP002954.1, AC007277.1, and AC008549.1) for the construction of a prognostic risk signature. We found that the survival in the low-risk group was better than that in the high-risk group, and the risk score model was reliable for predicting prognosis as an independent indicator of HCC. Stratification analyses revealed that the signature could serve as a prognostic marker for diverse populations of HCC, whether older or younger patients or early- or advanced-stage patients. In addition, the established nomogram based on the risk scores showed better clinical practicality than did the traditional tumor grade or clinical stage system.

Growing evidence highlights the importance of lncRNA in the cancer landscape, such as cell cycle, cell differentiation, DNA repair, epithelial–mesenchymal transition (EMT), and immune regulation (Hu et al., 2018). Some of the lncRNAs in this risk model have already been identified as involved in the malignant phenotypes of HCC or other cancers. For instance, LINC00426 was significantly upregulated in lung adenocarcinoma and played a notable role in accelerating tumor proliferation, invasion, metastasis, and EMT *in vitro* and *in vivo* (Li et al., 2020). LINC00426 may reshape the tumor immune microenvironment, which is positively associated with Th cell differentiation, cytokine signaling pathways, and multiple immune markers, including cytotoxic markers, co-inhibitory and co-stimulatory molecules (i.e., PD1, CTLA4, HAVCR2, TIGIT, FOXP3, and ICOS), and chemokine receptors and ligands (i.e., CXCR3/6, CXCL9/13, CCL4/5/7/19, and CCR7) (Wang et al., 2021b). LINC002273 was reported to be correlated with tumor proliferation, migration, and invasion by epigenetically increasing the AGR2 transcription in breast cancer (Xiu et al., 2019). Nevertheless, the other four prognostic TME-related lncRNAs (LINC01150, AP002954.1, AC007277.1, and AC008549.1) have been rarely reported. Thus, this TME-related lncRNA signature could identify novel biomarkers for further research. In addition, we found that the TME-related lncRNA risk signature was primarily involved in regulating DNA replication, cell cycle, ErbB signaling pathway, Notch signaling pathway, and immunologic function.

In recent years, ICIs have shown survival benefit and have been approved as first- or second-line therapies for HCC (Ntziachristos et al., 2014). Nevertheless, only a minority of patients with HCC can benefit from immune therapy, and less than 30% of patients have an objective response to ICIs. Thus, exploring more accurate biomarkers to forecast responsiveness to ICI treatment and further screening of the dominant population have become a major challenge in HCC. However, currently, there is no standard and effective biomarker that predicts the treatment response of HCC patients to ICIs (Li et al., 2019b). It was reported that a high PD-L1 expression on tumor cells was associated with better survival, but not predictive of the objective response rate (ORR) (Cho et al., 2009). A high PD-1 expression in tumor was associated with ORR (Cho et al., 2009; Sangro et al., 2020). Recently, pieces of evidence have indicated that some lncRNAs were involved in the regulation of ICI-related genes in the cancer immunity cycle (Tchekneva et al., 2019; Wu et al., 2020). For instance, the lncRNA AFAP1-AS1 has been discovered to regulate PD-1/PD-L1 signaling in the TME of nasopharyngeal carcinoma, and its expression was positively associated with PD-1 (Tang et al., 2017; Tchekneva et al., 2019). Previous studies reported that immune-related lncRNA signatures can predict the treatment response to ICIs, which focused on analyzing the association of lncRNAs and ICB-related key genes (*PD-1*, *PD-L1*, *PD-L2*, and *CTLA4*) (Zhang et al., 2020; Zhou et al., 2021b). In this study, we comprehensively explored the association among 46 immune checkpoint-related genes, IPS, and the TME-related lncRNA signature, with the aim of finding reliable predictors of ICI therapy in HCC. We revealed that 20 out of the 46 ICB-related genes were significantly different between the high- and low-risk groups, and the expression levels of *PD-L2* and *CTLA4* were significantly upregulated in the low-risk group. Furthermore, HCC patients in the low-risk group exhibited higher IPS and tended to have a better response to ICI treatment. These findings indicate that the prognostic TME-related lncRNA signature may be able to predict the clinical outcome of ICI therapy in HCC samples. In addition, increasing evidence has demonstrated that lncRNAs seriously affect immune cell infiltration (Wang et al., 2010). Thus, we analyzed the correlation between the TME-related lncRNA risk signature and specific immune cells or immune pathways. We discovered that some immune cells, which were beneficial for enhancing immune responses toward cancer, were remarkably upregulated in the low-risk group, including B cells, CD8^+^ T cells, mast cells, follicular Th cells, Th1 cells, and TILs. Moreover, some immune infiltration cells were negatively correlated with the risk score, such as those of naive B cells, activated memory CD4^+^ T cells, CD8^+^ T cells, and γδ T cells, which can be understood as, the higher the risk score, the lower the infiltration level of the immune response cells. Conversely, M2 macrophages were significantly positively correlated with the risk score, which contributes to tumor angiogenesis, metastasis, EMT, and immune suppression in HCC (Sierra et al., 2017). The differences in the immune cells between the high- and low-risk groups may partly account for HCC patients in the high-risk group showing worse prognosis than those in the low-risk group.

When comparing this study with previous studies that explored the immune-related biomolecular predictors in HCC, some advantages of our research are identified. Firstly, we focused on identifying novel prognostic lncRNA markers from the perspective of the TME. Secondly, we established a reasonable relationship between lncRNAs and the TME based on the intersecting DElncRNAs of the TME scores and constructed a reliable risk signature, rather than relying on immune-related genes. Finally, we comprehensively explored the association of the TME-related lncRNA signatures with the ICI response of HCC patients according to the ICI-related genes and IPS.

In conclusion, we performed an overall analysis of the association between the TME scores and survival of HCC patients. A novel TME-related prognostic risk signature was established, which could be effectively applied as an independent prognostic biomarker and a predictive factor of ICI therapy for HCC patients.

## Data Availability

The original contributions presented in the study are included in the article/Supplementary Material. Further inquiries can be directed to the corresponding author.

## Abbreviations

AIC, Akaike information criterion; AUC, area under the ROC curve; CI, confidence interval; CTLA4, cytotoxic T lymphocyte antigen 4; DElncRNAs, differentially expressed lncRNAs; DEGs, differentially expressed genes; DFS, disease-free survival; EGFR, epidermal growth factor receptor; FDR, false discovery rate; GO, Gene Ontology; GSEA, gene set enrichment analysis; HCC, hepatocellular carcinoma; HR, hazard ratio; ICIs, immune checkpoint inhibitors; IPS, immunophenoscore; LncRNAs, long noncoding RNAs; ORR, objective response rate; OS, overall survival; PD-1, programmed death-1; PD-L1, programmed death-ligand 1; PD-L2, programmed death-ligand 2; PFS, progression-free survival; *R*, Spearman’s correlation coefficient; ROC, receiver operating characteristic; ssGSEA, single-sample gene set enrichment analysis; TCGA, The Cancer Genome Atlas; TGF-β, transforming growth factor beta; TILs, tumor-infiltrating lymphocytes; TME, tumor microenvironment
