# Supplementary material for: Identification of Novel Tumor Microenvironment-Related Long Noncoding RNAs to Determine the Prognosis and Response to Immunotherapy of Hepatocellular Carcinoma Patients
Source: Front Mol Biosci. 2021 Dec 24;8:781307. doi: 10.3389/fmolb.2021.781307 (PMC8739902; doi:10.3389/fmolb.2021.781307)
Supplement: Supplementary file 1 [file Table1.DOCX]

Table S1. Stromal/Immune /Estimate scores of HCC patients based on the ESTIMATE algorithm

| id | Stromal Score | Immune Score | ESTIMATE Score |
| --- | --- | --- | --- |
| TCGA-DD-AAE4 | 50.22355 | 1773.539 | 1823.763 |
| TCGA-CC-A3M9 | 889.401 | 2833.527 | 3722.928 |
| TCGA-DD-A4NI | -126.538 | 1033.749 | 907.2108 |
| TCGA-G3-AAV3 | -316.236 | 1377.087 | 1060.85 |
| TCGA-UB-A7MA | -819.093 | -16.9756 | -836.068 |
| TCGA-UB-A7MF | -605.438 | 1820.298 | 1214.86 |
| TCGA-BC-A112 | -283.328 | 466.3386 | 183.011 |
| TCGA-BC-A10U | -1251.87 | 69.17789 | -1182.69 |
| TCGA-BC-A5W4 | -1450.47 | -629.581 | -2080.05 |
| TCGA-FV-A3I1 | -341.786 | 894.0664 | 552.2807 |
| TCGA-CC-A123 | -850.928 | 377.6595 | -473.269 |
| TCGA-WQ-A9G7 | -1276.46 | -256.357 | -1532.82 |
| TCGA-ED-A66X | 326.1796 | 936.4772 | 1262.657 |
| TCGA-DD-A1EK | -359.011 | 818.2729 | 459.2614 |
| TCGA-DD-AADM | -575.126 | 535.1757 | -39.9506 |
| TCGA-MR-A8JO | 759.9058 | 1464.55 | 2224.455 |
| TCGA-QA-A7B7 | -1327.08 | -36.6027 | -1363.69 |
| TCGA-CC-A8HT | -1053.38 | 824.831 | -228.547 |
| TCGA-FV-A4ZP | -731.267 | 1235.37 | 504.1027 |
| TCGA-DD-A4NJ | -190.803 | 856.219 | 665.4157 |
| TCGA-CC-5258 | -559.62 | 628.4768 | 68.85733 |
| TCGA-EP-A2KB | -1120.22 | -10.4399 | -1130.66 |
| TCGA-G3-A25X | -81.3593 | 1502.131 | 1420.772 |
| TCGA-DD-AAD1 | 266.8656 | 1366.649 | 1633.515 |
| TCGA-G3-A3CK | -700.08 | 721.7696 | 21.68975 |
| TCGA-DD-AACA | -1119.91 | -515.201 | -1635.11 |
| TCGA-5C-A9VH | -296.797 | 76.23914 | -220.557 |
| TCGA-FV-A23B | -661.588 | 497.1231 | -164.464 |
| TCGA-DD-AADR | -815.315 | 563.5625 | -251.752 |
| TCGA-DD-A4NQ | -953.375 | 637.5233 | -315.851 |
| TCGA-G3-A5SM | 74.61221 | 996.8552 | 1071.467 |
| TCGA-MI-A75E | -147.38 | 946.1324 | 798.7526 |
| TCGA-DD-A11D | -653.161 | 54.28381 | -598.877 |
| TCGA-CC-5262 | 232.0584 | 1187.982 | 1420.04 |
| TCGA-2Y-A9GY | -947.146 | 1759.101 | 811.9542 |
| TCGA-ES-A2HT | -209.201 | 737.8564 | 528.6558 |
| TCGA-DD-AAW0 | -6.9787 | 1097.722 | 1090.743 |
| TCGA-G3-A3CI | -336.857 | 976.3994 | 639.5428 |
| TCGA-DD-AAVV | 67.61635 | 1464.403 | 1532.019 |
| TCGA-DD-A39Y | -1194.49 | 28.29858 | -1166.19 |
| TCGA-DD-A1EB | -577.367 | 122.2266 | -455.141 |
| TCGA-DD-AAD6 | -1434.83 | 68.18206 | -1366.65 |
| TCGA-ED-A4XI | 192.5756 | 1156.853 | 1349.429 |
| TCGA-HP-A5MZ | 615.3122 | 1554.39 | 2169.702 |
| TCGA-DD-AAVU | -1181.3 | 25.94012 | -1155.35 |
| TCGA-DD-A4NO | -358.376 | 180.6288 | -177.748 |
| TCGA-BC-A10W | -622.098 | 488.7362 | -133.362 |
| TCGA-LG-A9QC | -952.479 | -374.507 | -1326.99 |
| TCGA-DD-A11C | -106.048 | 473.3442 | 367.2961 |
| TCGA-DD-AADB | -877.994 | 1230.553 | 352.5586 |
| TCGA-DD-AAVZ | -787.42 | 107.251 | -680.169 |
| TCGA-BC-A216 | -980.912 | 876.4525 | -104.459 |
| TCGA-DD-A1EF | -644.071 | 614.0772 | -29.9941 |
| TCGA-DD-AADL | -1221.16 | -319.67 | -1540.83 |
| TCGA-KR-A7K8 | -182.549 | 1629.427 | 1446.877 |
| TCGA-ED-A7PX | 37.18491 | 399.3103 | 436.4952 |
| TCGA-DD-AADK | -202.18 | 808.4494 | 606.2694 |
| TCGA-BW-A5NO | -685.723 | 531.074 | -154.649 |
| TCGA-DD-A39W | -982.302 | -41.8145 | -1024.12 |
| TCGA-GJ-A9DB | -213.798 | 358.6429 | 144.8447 |
| TCGA-DD-A1EA | -901.227 | 721.6426 | -179.584 |
| TCGA-UB-A7MD | -250.289 | 944.7586 | 694.4691 |
| TCGA-ES-A2HS | -492.395 | 10.43447 | -481.96 |
| TCGA-G3-A3CJ | -804.45 | 257.9042 | -546.546 |
| TCGA-FV-A3R2 | -1079.61 | 142.6949 | -936.917 |
| TCGA-2Y-A9GZ | -995.163 | -382.13 | -1377.29 |
| TCGA-4R-AA8I | -735.952 | 190.8571 | -545.095 |
| TCGA-DD-A4NG | -839.335 | 482.6824 | -356.653 |
| TCGA-RC-A6M5 | 342.4549 | 233.0595 | 575.5144 |
| TCGA-2Y-A9H0 | -1069.74 | 810.5094 | -259.232 |
| TCGA-DD-A11B | -865.844 | -292.68 | -1158.52 |
| TCGA-G3-A25V | 53.51848 | 1168.653 | 1222.172 |
| TCGA-5R-AA1C | -803.632 | 353.5736 | -450.058 |
| TCGA-ED-A8O6 | -946.346 | 17.65268 | -928.694 |
| TCGA-DD-AADD | -1476.24 | -89.1235 | -1565.36 |
| TCGA-BC-A10S | 37.27716 | 1167.347 | 1204.624 |
| TCGA-DD-AAEB | -766.297 | 400.3097 | -365.987 |
| TCGA-WX-AA44 | -911.847 | 393.606 | -518.241 |
| TCGA-PD-A5DF | 161.8668 | 899.5525 | 1061.419 |
| TCGA-YA-A8S7 | 136.7208 | 1793.695 | 1930.416 |
| TCGA-5R-AA1D | 1180.26 | 1039.202 | 2219.463 |
| TCGA-DD-AACH | -749.611 | 365.4137 | -384.197 |
| TCGA-CC-A8HV | -1243.76 | -407.45 | -1651.21 |
| TCGA-G3-AAV0 | -816.413 | 247.9202 | -568.493 |
| TCGA-DD-AAED | -1520.69 | 182.5625 | -1338.12 |
| TCGA-DD-A73F | -158.285 | 1796.204 | 1637.919 |
| TCGA-DD-A4NN | -839.888 | -400.387 | -1240.28 |
| TCGA-DD-AAEI | -641.648 | 754.1842 | 112.5364 |
| TCGA-UB-AA0V | 129.5588 | 626.6908 | 756.2496 |
| TCGA-DD-AADO | -779.208 | 1464.276 | 685.0672 |
| TCGA-DD-AAVY | -1164.22 | -280.528 | -1444.75 |
| TCGA-FV-A3R3 | 428.4665 | 822.8799 | 1251.346 |
| TCGA-DD-A4NS | 587.01 | 1243.042 | 1830.052 |
| TCGA-MI-A75H | -529.733 | 1061.121 | 531.3881 |
| TCGA-UB-A7MC | -1327.81 | -468.168 | -1795.98 |
| TCGA-FV-A4ZQ | -966.426 | 1757.74 | 791.3137 |
| TCGA-DD-AAEG | -998.087 | -219.42 | -1217.51 |
| TCGA-DD-AAD5 | -951.955 | 837.4232 | -114.531 |
| TCGA-K7-A5RG | -208.962 | 2481.757 | 2272.796 |
| TCGA-XR-A8TD | 401.2969 | 1913.655 | 2314.952 |
| TCGA-DD-AACY | -747.463 | 333.7371 | -413.726 |
| TCGA-KR-A7K7 | -1100.33 | 1209.631 | 109.2997 |
| TCGA-G3-A25Y | -181.154 | 605.8051 | 424.6508 |
| TCGA-K7-AAU7 | -271.021 | 666.8142 | 395.7935 |
| TCGA-BC-A3KF | -827.787 | -77.8315 | -905.619 |
| TCGA-BC-A10Z | -1472.79 | -722.562 | -2195.35 |
| TCGA-UB-AA0U | 41.75402 | 766.0743 | 807.8283 |
| TCGA-DD-AAEE | -971.933 | -216.736 | -1188.67 |
| TCGA-ZP-A9D0 | -725.631 | -179.06 | -904.691 |
| TCGA-DD-A1EG | -15.5715 | 1393.334 | 1377.762 |
| TCGA-CC-A7IL | -1544.81 | -454.136 | -1998.94 |
| TCGA-RC-A6M4 | -1265.38 | -636.683 | -1902.06 |
| TCGA-ED-A7XP | -347.168 | 511.9592 | 164.7915 |
| TCGA-2Y-A9HA | -1002.41 | -34.4823 | -1036.89 |
| TCGA-DD-AAE2 | -233.675 | 934.5449 | 700.8699 |
| TCGA-DD-AADF | -1152.74 | 313.1818 | -839.561 |
| TCGA-DD-AACW | -1105.05 | 178.6623 | -926.39 |
| TCGA-MI-A75I | -1152.91 | 216.8422 | -936.071 |
| TCGA-FV-A3I0 | -460.09 | 366.3987 | -93.6913 |
| TCGA-2Y-A9H1 | -853.011 | 1138.137 | 285.1258 |
| TCGA-CC-A8HU | -1191.28 | -220 | -1411.28 |
| TCGA-DD-AAD2 | 279.2252 | 1019.556 | 1298.781 |
| TCGA-BC-A10R | -100.377 | 19.32305 | -81.054 |
| TCGA-G3-A5SL | -665.528 | -41.9445 | -707.473 |
| TCGA-CC-A1HT | 162.9672 | 2106.489 | 2269.457 |
| TCGA-2Y-A9GV | -120.295 | 901.7901 | 781.4948 |
| TCGA-ED-A7XO | -154.07 | 803.4127 | 649.3429 |
| TCGA-CC-A5UC | -263.718 | 412.5811 | 148.8635 |
| TCGA-DD-AAVQ | -298.721 | 2022.506 | 1723.785 |
| TCGA-BD-A3EP | 163.7083 | 1482.001 | 1645.709 |
| TCGA-DD-A39V | -389.295 | 1389.999 | 1000.704 |
| TCGA-DD-A3A1 | -1023.86 | 914.8023 | -109.058 |
| TCGA-DD-A4NR | 163.0175 | 2304.798 | 2467.816 |
| TCGA-DD-AAC9 | 163.6089 | 1137.337 | 1300.946 |
| TCGA-CC-5259 | -1298.13 | 590.5668 | -707.564 |
| TCGA-DD-AADC | -1095.42 | -99.859 | -1195.28 |
| TCGA-ZS-A9CF | -860.624 | 273.7069 | -586.917 |
| TCGA-NI-A8LF | -380.63 | 80.98753 | -299.642 |
| TCGA-DD-A39Z | -1365.24 | -192.663 | -1557.9 |
| TCGA-G3-A7M5 | -944.066 | 377.7936 | -566.272 |
| TCGA-MI-A75G | -1088.23 | -274.031 | -1362.26 |
| TCGA-CC-A7IK | -1300.14 | -553.25 | -1853.39 |
| TCGA-G3-A5SJ | -591.584 | 1251.424 | 659.8402 |
| TCGA-ZS-A9CE | -1195.64 | -498.65 | -1694.29 |
| TCGA-DD-A73B | -1148.02 | -347.355 | -1495.38 |
| TCGA-RC-A7SB | -1046.39 | 47.24046 | -999.153 |
| TCGA-DD-AADN | -1103.8 | 2010.582 | 906.785 |
| TCGA-CC-A5UE | -1508.5 | 6.053282 | -1502.45 |
| TCGA-DD-AACZ | -449.581 | 2057.853 | 1608.272 |
| TCGA-DD-A3A3 | -1358.33 | 299.4537 | -1058.88 |
| TCGA-2Y-A9H8 | -919.535 | 931.9993 | 12.46469 |
| TCGA-DD-AAEK | -171.074 | 1529.34 | 1358.266 |
| TCGA-DD-A4ND | 618.3545 | 1158.677 | 1777.031 |
| TCGA-2Y-A9H7 | -1174.76 | 335.0884 | -839.675 |
| TCGA-RC-A7S9 | -1177.5 | 546.7834 | -630.716 |
| TCGA-G3-A25S | -1022.57 | 659.2328 | -363.339 |
| TCGA-BC-A217 | -1113.59 | 705.9782 | -407.616 |
| TCGA-DD-AAW2 | -814.426 | -234.241 | -1048.67 |
| TCGA-2Y-A9H6 | -22.4783 | 1079.278 | 1056.8 |
| TCGA-2Y-A9GW | 32.85979 | 1487.878 | 1520.738 |
| TCGA-DD-A73D | -1521.78 | -649.891 | -2171.67 |
| TCGA-DD-A115 | -333.94 | 467.2478 | 133.3076 |
| TCGA-XR-A8TF | -1093.79 | -98.3382 | -1192.13 |
| TCGA-DD-AADU | -1270.19 | 12.55331 | -1257.64 |
| TCGA-DD-AAE1 | -1133.6 | 349.5315 | -784.067 |
| TCGA-2Y-A9HB | -237.399 | 963.1797 | 725.7808 |
| TCGA-BD-A2L6 | -680.77 | 419.8323 | -260.938 |
| TCGA-DD-A3A9 | -61.194 | 880.1284 | 818.9344 |
| TCGA-KR-A7K2 | -379.953 | 604.1275 | 224.1748 |
| TCGA-ED-A66Y | -1223.73 | -823.557 | -2047.29 |
| TCGA-DD-AAE3 | -191.232 | 423.7321 | 232.4998 |
| TCGA-G3-AAV6 | -1079.59 | -314.765 | -1394.35 |
| TCGA-ED-A97K | -351.876 | 541.3542 | 189.4779 |
| TCGA-DD-AAVW | 226.6671 | 822.5244 | 1049.191 |
| TCGA-EP-A3RK | -99.5973 | 1774.389 | 1674.792 |
| TCGA-FV-A496 | -1302.62 | -557.39 | -1860.01 |
| TCGA-CC-A3MB | -962.873 | 41.4814 | -921.392 |
| TCGA-CC-A7IF | -1095.6 | -334.965 | -1430.56 |
| TCGA-ZS-A9CD | -259.447 | 674.2278 | 414.7812 |
| TCGA-G3-A7M6 | -650.456 | 278.0143 | -372.442 |
| TCGA-DD-A1ED | -91.051 | 1142.394 | 1051.343 |
| TCGA-EP-A12J | -853.535 | 54.08707 | -799.448 |
| TCGA-DD-A1EJ | -1034.1 | 304.6916 | -729.407 |
| TCGA-G3-AAV2 | -782.932 | 96.88943 | -686.043 |
| TCGA-DD-AADY | -662.031 | -388.568 | -1050.6 |
| TCGA-CC-5263 | -792.122 | 645.9539 | -146.168 |
| TCGA-DD-AADP | -523.599 | 386.7913 | -136.808 |
| TCGA-BC-A69I | -262.38 | 490.4278 | 228.0482 |
| TCGA-5C-A9VG | -421.396 | -291.003 | -712.399 |
| TCGA-DD-A1EE | -1035.92 | -223.432 | -1259.35 |
| TCGA-BC-A110 | 660.9759 | 1589.133 | 2250.109 |
| TCGA-GJ-A3OU | 925.0539 | 2610.54 | 3535.594 |
| TCGA-G3-A25U | -1080.74 | -210.495 | -1291.23 |
| TCGA-DD-A4NL | -208.16 | 420.3637 | 212.2041 |
| TCGA-BC-A10Q | -683.751 | 221.6059 | -462.145 |
| TCGA-2Y-A9GU | -1010.13 | -174.713 | -1184.84 |
| TCGA-ZP-A9CV | -150.597 | 956.5058 | 805.9086 |
| TCGA-DD-A113 | -722.694 | 511.5984 | -211.095 |
| TCGA-2Y-A9GT | -164.481 | 1517.792 | 1353.311 |
| TCGA-CC-A3MC | -545.462 | 343.4599 | -202.002 |
| TCGA-DD-A73A | -894.688 | 871.5611 | -23.1269 |
| TCGA-DD-AADG | -1047.73 | -395.835 | -1443.56 |
| TCGA-FV-A2QR | -233.547 | 217.2224 | -16.3242 |
| TCGA-G3-AAV7 | -665.42 | 834.8466 | 169.4269 |
| TCGA-2Y-A9H4 | -779.011 | 585.8781 | -193.133 |
| TCGA-3K-AAZ8 | -1024 | 444.7799 | -579.216 |
| TCGA-CC-5261 | 69.8345 | 1125.863 | 1195.698 |
| TCGA-ZP-A9D2 | -515.622 | 252.807 | -262.815 |
| TCGA-2Y-A9H5 | -309.108 | 283.4464 | -25.6613 |
| TCGA-G3-A25T | -454.822 | 357.646 | -97.1755 |
| TCGA-DD-AAEA | -1008.4 | 716.9278 | -291.474 |
| TCGA-RG-A7D4 | -1092.41 | 1537.178 | 444.7669 |
| TCGA-DD-AACD | -384.398 | 1065.898 | 681.4999 |
| TCGA-DD-AAD8 | -968.521 | 135.8593 | -832.661 |
| TCGA-5R-AAAM | 523.2681 | 1113.124 | 1636.392 |
| TCGA-EP-A3JL | 1.250757 | 1360.955 | 1362.206 |
| TCGA-DD-A73G | -703.279 | 622.5973 | -80.6812 |
| TCGA-G3-AAV4 | -922.353 | -88.2295 | -1010.58 |
| TCGA-ED-A459 | -854.962 | 558.7397 | -296.223 |
| TCGA-DD-A73C | -125.128 | 253.3792 | 128.2515 |
| TCGA-MR-A520 | -739.751 | 217.6285 | -522.122 |
| TCGA-DD-AACU | -690.904 | 440.5014 | -250.403 |
| TCGA-WX-AA47 | -1379.19 | -401.421 | -1780.61 |
| TCGA-DD-A3A4 | -867.242 | -440.326 | -1307.57 |
| TCGA-DD-AADQ | -1160.11 | 549.6183 | -610.496 |
| TCGA-CC-A8HS | -1160.66 | -387.746 | -1548.41 |
| TCGA-DD-A1EL | -1299.03 | 227.117 | -1071.92 |
| TCGA-WQ-AB4B | -260.243 | 638.0469 | 377.8042 |
| TCGA-DD-A11A | -1054.6 | -191.146 | -1245.75 |
| TCGA-DD-AAD0 | -1419.04 | -235.822 | -1654.86 |
| TCGA-DD-A3A6 | 679.1427 | 1306.978 | 1986.121 |
| TCGA-DD-A114 | 281.4384 | 1812.561 | 2093.999 |
| TCGA-5C-AAPD | -261.275 | 1730.571 | 1469.296 |
| TCGA-HP-A5N0 | 172.466 | 575.3598 | 747.8258 |
| TCGA-T1-A6J8 | -510.192 | 209.2138 | -300.978 |
| TCGA-XR-A8TC | -804.77 | -209.435 | -1014.2 |
| TCGA-DD-AACK | -1057.86 | 152.477 | -905.38 |
| TCGA-MI-A75C | -1437.68 | 632.1407 | -805.539 |
| TCGA-DD-AACP | -1622.33 | -286.644 | -1908.97 |
| TCGA-DD-AACT | -356.164 | 966.9819 | 610.8183 |
| TCGA-DD-A73E | -1162.69 | -760.213 | -1922.9 |
| TCGA-BC-4072 | -328.382 | 1390.231 | 1061.849 |
| TCGA-BC-4073 | -17.7901 | 1927.226 | 1909.436 |
| TCGA-XR-A8TG | -483.401 | 299.1962 | -184.205 |
| TCGA-RC-A6M6 | -1395.4 | -2.05092 | -1397.45 |
| TCGA-BC-A69H | -811.647 | 932.2165 | 120.5694 |
| TCGA-DD-AAC8 | -914.615 | 425.3469 | -489.268 |
| TCGA-DD-AADJ | -1006.26 | 471.7245 | -534.538 |
| TCGA-2Y-A9H9 | -675.659 | 342.8331 | -332.826 |
| TCGA-ED-A5KG | 560.7323 | 3157.283 | 3718.016 |
| TCGA-DD-AACO | -480.065 | 935.0023 | 454.9374 |
| TCGA-G3-A25Z | -463.486 | 453.6376 | -9.84835 |
| TCGA-DD-A4NV | -221.97 | 257.4978 | 35.52767 |
| TCGA-DD-A39X | -436.833 | 571.272 | 134.4393 |
| TCGA-EP-A2KA | -770.978 | 1462.072 | 691.0933 |
| TCGA-DD-A1EC | -180.516 | 2424.078 | 2243.562 |
| TCGA-DD-AAD3 | 21.89845 | 720.262 | 742.1604 |
| TCGA-DD-AACS | -1224.78 | -341.527 | -1566.31 |
| TCGA-BD-A3ER | 92.45868 | 619.3027 | 711.7614 |
| TCGA-GJ-A6C0 | -542.935 | 1112.671 | 569.736 |
| TCGA-2Y-A9GS | -524.653 | 636.3562 | 111.7034 |
| TCGA-BC-A8YO | -898.157 | 1213.703 | 315.5455 |
| TCGA-CC-A9FW | -1331.77 | 171.0726 | -1160.7 |
| TCGA-CC-A5UD | -1160.69 | -202.896 | -1363.58 |
| TCGA-DD-A119 | -349.155 | 355.2854 | 6.130247 |
| TCGA-DD-AACB | -680.741 | 1433.362 | 752.6207 |
| TCGA-FV-A495 | 66.13022 | 1334.623 | 1400.753 |
| TCGA-DD-A1EH | -484.884 | -91.026 | -575.91 |
| TCGA-DD-AAEH | -187.803 | 1486.344 | 1298.541 |
| TCGA-DD-A4NE | -719.127 | -220.439 | -939.566 |
| TCGA-DD-AADV | -427.956 | 182.6944 | -245.261 |
| TCGA-DD-AACX | -1259.62 | 203.3218 | -1056.3 |
| TCGA-DD-AACC | 133.8146 | 2617.594 | 2751.408 |
| TCGA-G3-A5SK | -295.818 | 1011.697 | 715.8787 |
| TCGA-DD-AACV | -1389.19 | -710.032 | -2099.22 |
| TCGA-CC-5264 | -992.398 | 82.68512 | -909.713 |
| TCGA-G3-A7M7 | -798.427 | -46.8864 | -845.313 |
| TCGA-G3-AAUZ | -374.777 | 1046.63 | 671.853 |
| TCGA-RC-A7SK | -1155.01 | -346.13 | -1501.14 |
| TCGA-DD-AACI | -155.025 | 1870.706 | 1715.681 |
| TCGA-DD-A4NH | -617.975 | 935.2918 | 317.3164 |
| TCGA-WX-AA46 | -284.135 | 444.5405 | 160.4053 |
| TCGA-2Y-A9H2 | -983.096 | 589.4897 | -393.606 |
| TCGA-DD-AAW3 | -916.884 | -302.301 | -1219.18 |
| TCGA-ED-A82E | -389.482 | 269.4677 | -120.014 |
| TCGA-DD-A4NP | -715.576 | 208.5113 | -507.065 |
| TCGA-G3-A6UC | -1162.77 | -263.132 | -1425.9 |
| TCGA-FV-A2QQ | -449.096 | 876.18 | 427.0836 |
| TCGA-ZP-A9D4 | -963.81 | -208.533 | -1172.34 |
| TCGA-DD-AAE6 | -1603.81 | -861.772 | -2465.59 |
| TCGA-DD-AACG | -1197.81 | 783.8553 | -413.952 |
| TCGA-G3-A5SI | -1042.96 | -481.116 | -1524.08 |
| TCGA-ED-A7PY | -1312.62 | -233.811 | -1546.43 |
| TCGA-EP-A2KC | -799.158 | 148.582 | -650.576 |
| TCGA-BC-A10X | 357.0137 | 870.5171 | 1227.531 |
| TCGA-CC-A7IH | -970.249 | -439.413 | -1409.66 |
| TCGA-DD-A3A8 | -1103.95 | -157.809 | -1261.76 |
| TCGA-DD-AACL | -490.185 | 1787.774 | 1297.589 |
| TCGA-DD-A116 | -632.489 | 459.2291 | -173.259 |
| TCGA-CC-A7IG | -946.161 | 482.1818 | -463.979 |
| TCGA-DD-AAE9 | -1369.22 | 27.59223 | -1341.63 |
| TCGA-DD-AAVP | -621.256 | 112.0546 | -509.201 |
| TCGA-G3-AAV5 | -1093.93 | 98.15718 | -995.77 |
| TCGA-G3-A3CG | -420.833 | 462.0498 | 41.21727 |
| TCGA-CC-5260 | 56.84604 | 699.3841 | 756.2302 |
| TCGA-CC-A7IJ | 838.7786 | 1934.96 | 2773.738 |
| TCGA-DD-A4NK | -716.5 | -80.7816 | -797.281 |
| TCGA-DD-A3A5 | -652.856 | 127.7541 | -525.101 |
| TCGA-NI-A4U2 | -465.599 | 205.1918 | -260.407 |
| TCGA-DD-A4NF | -843.938 | -409.499 | -1253.44 |
| TCGA-DD-AADI | -177.249 | 1587.159 | 1409.91 |
| TCGA-DD-A4NA | -407.584 | 583.0748 | 175.4912 |
| TCGA-BC-A10T | 133.0674 | 214.3257 | 347.3931 |
| TCGA-RC-A7SH | -1244.02 | -387.769 | -1631.79 |
| TCGA-WJ-A86L | -1359.92 | -136.199 | -1496.12 |
| TCGA-DD-AA3A | -1464.45 | -28.2263 | -1492.67 |
| TCGA-CC-A7IE | -656.41 | 1255.066 | 598.6565 |
| TCGA-DD-AAVS | -682.421 | 223.0969 | -459.324 |
| TCGA-DD-AAE7 | -221.023 | 888.652 | 667.6293 |
| TCGA-BC-A3KG | -1278.58 | -90.1274 | -1368.71 |
| TCGA-DD-A3A2 | -558.85 | 182.5098 | -376.34 |
| TCGA-DD-AAVR | 390.8852 | 1716.209 | 2107.094 |
| TCGA-CC-A7II | -764.256 | -328.885 | -1093.14 |
| TCGA-DD-AACN | -304.391 | 364.6451 | 60.25447 |
| TCGA-2Y-A9GX | 730.6225 | 1703.763 | 2434.386 |
| TCGA-CC-A3MA | -486.846 | -78.8242 | -565.67 |
| TCGA-DD-AACQ | -1477.56 | -532.04 | -2009.6 |
| TCGA-RC-A7SF | -966.822 | 322.6571 | -644.164 |
| TCGA-DD-AADW | -540.44 | -353.928 | -894.368 |
| TCGA-G3-A3CH | -425.278 | 604.8997 | 179.6219 |
| TCGA-DD-A1EI | 1.053466 | 529.3055 | 530.359 |
| TCGA-ZP-A9CZ | -235.682 | 1158.532 | 922.8494 |
| TCGA-KR-A7K0 | -529.473 | 314.6548 | -214.819 |
| TCGA-BC-A10Y | -830.616 | 454.6962 | -375.919 |
| TCGA-ED-A8O5 | -709.619 | -178.753 | -888.372 |
| TCGA-CC-A9FS | -949.396 | 31.3563 | -918.039 |
| TCGA-UB-A7MB | -1250.65 | -458.507 | -1709.15 |
| TCGA-ED-A627 | 734.8166 | 1923.451 | 2658.267 |
| TCGA-XR-A8TE | -527.753 | 17.66269 | -510.091 |
| TCGA-DD-AACF | -988.157 | 712.9044 | -275.253 |
| TCGA-K7-A5RF | 299.7977 | 1357.09 | 1656.887 |
| TCGA-ZP-A9D1 | -203.065 | 657.4466 | 454.3816 |
| TCGA-DD-A3A7 | -1115.29 | 322.6387 | -792.656 |
| TCGA-DD-AACE | -719.284 | -55.8487 | -775.132 |
| TCGA-2Y-A9H3 | -156.446 | 1790.491 | 1634.045 |
| TCGA-DD-A4NB | 1006.761 | 893.853 | 1900.614 |
| TCGA-EP-A26S | -896.75 | -289.61 | -1186.36 |
| TCGA-DD-AAVX | -481.201 | 978.0979 | 496.897 |
| TCGA-ED-A7PZ | -1482.06 | -510.339 | -1992.39 |
| TCGA-LG-A6GG | -1087.13 | -71.4084 | -1158.54 |
| TCGA-UB-A7ME | -418.69 | 1198.352 | 779.6617 |
| TCGA-G3-A7M9 | -1351.16 | -336.592 | -1687.75 |
| TCGA-DD-AACJ | -1254.31 | -13.5851 | -1267.89 |
| TCGA-LG-A9QD | -769.875 | 509.0336 | -260.841 |
| TCGA-ZS-A9CG | -727.744 | 109.7798 | -617.964 |
| TCGA-K7-A6G5 | -355.707 | 861.5631 | 505.8557 |
| TCGA-DD-AADS | -698.598 | 1432.598 | 733.9994 |
| TCGA-DD-AAW1 | -927.093 | -127.434 | -1054.53 |
| TCGA-DD-A118 | -370.057 | 54.17269 | -315.884 |
| TCGA-DD-AADA | -290.881 | 1053.569 | 762.6877 |
| TCGA-G3-AAV1 | -769.709 | -32.0374 | -801.746 |
| TCGA-O8-A75V | -224.245 | 1192.89 | 968.6458 |
| TCGA-ZP-A9CY | -83.7052 | 639.0403 | 555.335 |
| TCGA-G3-A7M8 | -283.261 | 309.8199 | 26.5592 |
| TCGA-DD-AAE0 | -656.286 | 67.73899 | -588.547 |
