# Supplementary material for: Identification of Novel Tumor Microenvironment-Related Long Noncoding RNAs to Determine the Prognosis and Response to Immunotherapy of Hepatocellular Carcinoma Patients
Source: Front Mol Biosci. 2021 Dec 24;8:781307. doi: 10.3389/fmolb.2021.781307 (PMC8739902; doi:10.3389/fmolb.2021.781307)
Supplement: Supplementary file 2 [file Table2.DOCX]

Table S2. Differential expression lncRNAs were identified in the stromal scores group

| **Gene** | **Low stromal scores group** | **High stromal scores group** | **logFC** | **pValue** | **FDR** |
| --- | --- | --- | --- | --- | --- |
| AC005550.2 | 0.642173 | 0.13503 | -2.24968 | 0.000966 | 0.002542 |
| LINC02587 | 0.49327 | 0.108761 | -2.18121 | 7.62E-06 | 3.73E-05 |
| AC104088.1 | 0.298478 | 0.0713 | -2.06566 | 4.74E-06 | 2.47E-05 |
| LINC01139 | 1.406153 | 0.356139 | -1.98124 | 0.001522 | 0.003753 |
| CASC22 | 0.250979 | 0.064317 | -1.96429 | 0.000388 | 0.001172 |
| LINC02413 | 0.516685 | 0.133813 | -1.94906 | 0.007395 | 0.01472 |
| AL355987.4 | 0.421415 | 0.112164 | -1.90963 | 9.05E-07 | 5.51E-06 |
| AC007277.1 | 0.205133 | 0.055212 | -1.89349 | 8.38E-07 | 5.18E-06 |
| AC026765.2 | 1.322021 | 0.370048 | -1.83696 | 5.12E-06 | 2.64E-05 |
| AC239809.3 | 0.185191 | 0.054711 | -1.75911 | 0.002484 | 0.005793 |
| AC079305.1 | 0.899313 | 0.266768 | -1.75324 | 7.16E-05 | 0.000267 |
| AC011747.1 | 0.295056 | 0.090456 | -1.70569 | 3.22E-05 | 0.000131 |
| LINC02241 | 1.359261 | 0.417981 | -1.70131 | 5.90E-06 | 2.98E-05 |
| LINC01970 | 0.194295 | 0.060524 | -1.68268 | 3.48E-10 | 5.21E-09 |
| AL163953.1 | 0.597993 | 0.187167 | -1.6758 | 2.68E-10 | 4.14E-09 |
| AL590483.2 | 0.365317 | 0.114776 | -1.67032 | 0.000102 | 0.000363 |
| AC006205.2 | 15.53114 | 4.938571 | -1.653 | 0.003204 | 0.007199 |
| MIR325HG | 0.368121 | 0.126897 | -1.53653 | 0.000232 | 0.000743 |
| PART1 | 0.278185 | 0.097662 | -1.51017 | 0.00217 | 0.005155 |
| AC113404.1 | 0.54773 | 0.196158 | -1.48145 | 2.22E-09 | 2.63E-08 |
| LINC01124 | 5.019931 | 1.808886 | -1.47257 | 1.48E-11 | 2.94E-10 |
| AC007406.2 | 1.746323 | 0.633574 | -1.46273 | 4.96E-06 | 2.57E-05 |
| AC069294.1 | 0.552526 | 0.202773 | -1.44617 | 0.000506 | 0.001455 |
| LINC02315 | 1.037323 | 0.389992 | -1.41135 | 0.000226 | 0.000728 |
| AC010501.1 | 0.263305 | 0.099276 | -1.40722 | 3.03E-11 | 5.52E-10 |
| LINC02163 | 1.48276 | 0.559812 | -1.40527 | 6.87E-08 | 5.52E-07 |
| AP000593.3 | 2.172403 | 0.820907 | -1.404 | 0.003549 | 0.00789 |
| LINC01419 | 12.84879 | 4.896519 | -1.3918 | 0.016519 | 0.029513 |
| AC104123.1 | 0.157301 | 0.060068 | -1.38885 | 0.000134 | 0.000455 |
| KCNMB2-AS1 | 1.082395 | 0.416047 | -1.37941 | 0.000613 | 0.001721 |
| AC008549.1 | 16.32662 | 6.299495 | -1.37392 | 1.28E-05 | 5.88E-05 |
| AC016405.3 | 1.340219 | 0.518284 | -1.37065 | 1.71E-09 | 2.07E-08 |
| C5orf66 | 0.228368 | 0.088686 | -1.36458 | 0.013014 | 0.024027 |
| LINC00355 | 0.449855 | 0.175192 | -1.36053 | 8.03E-05 | 0.000296 |
| AL121845.4 | 2.981486 | 1.165964 | -1.35451 | 4.99E-12 | 1.12E-10 |
| LINC01980 | 2.529289 | 1.000247 | -1.33838 | 0.000612 | 0.001721 |
| AL139023.1 | 0.602432 | 0.238945 | -1.33412 | 0.000488 | 0.001415 |
| AC079062.1 | 0.543553 | 0.217122 | -1.32392 | 0.004184 | 0.00911 |
| LINC02506 | 3.421045 | 1.380625 | -1.30912 | 0.002303 | 0.005435 |
| AC110285.6 | 0.866118 | 0.354391 | -1.28922 | 9.40E-09 | 9.33E-08 |
| CASC9 | 3.370785 | 1.390695 | -1.27728 | 0.000521 | 0.001493 |
| LINC01952 | 0.259873 | 0.107633 | -1.27169 | 4.47E-09 | 4.75E-08 |
| BX649601.1 | 0.706106 | 0.30032 | -1.23338 | 1.12E-12 | 2.86E-11 |
| AC090150.1 | 4.148986 | 1.768384 | -1.23033 | 1.51E-05 | 6.73E-05 |
| SNHG25 | 7.026569 | 3.010668 | -1.22274 | 3.38E-08 | 2.90E-07 |
| AC093895.1 | 0.284343 | 0.122376 | -1.21631 | 0.022315 | 0.038122 |
| AC231981.1 | 0.591522 | 0.255029 | -1.21377 | 3.56E-09 | 3.97E-08 |
| LINC01194 | 0.402564 | 0.175649 | -1.19653 | 0.000692 | 0.001911 |
| AC132938.1 | 0.639998 | 0.28011 | -1.19207 | 0.010775 | 0.020284 |
| AC011445.2 | 3.898573 | 1.712622 | -1.18674 | 3.34E-05 | 0.000135 |
| AC010531.5 | 0.52019 | 0.229177 | -1.18257 | 0.00033 | 0.001017 |
| AC015849.5 | 0.171057 | 0.075519 | -1.17956 | 0.000317 | 0.000982 |
| AC124067.4 | 0.575792 | 0.255505 | -1.1722 | 0.001163 | 0.00298 |
| AL023583.1 | 0.248051 | 0.110109 | -1.17171 | 4.97E-06 | 2.57E-05 |
| FOXP4-AS1 | 0.596534 | 0.26586 | -1.16594 | 0.000838 | 0.002244 |
| AC091133.3 | 0.325144 | 0.145353 | -1.16152 | 0.006733 | 0.013564 |
| LINC00886 | 1.590349 | 0.71139 | -1.16063 | 1.81E-05 | 7.87E-05 |
| AC116049.2 | 0.574709 | 0.257252 | -1.15965 | 2.68E-09 | 3.11E-08 |
| LINC02475 | 0.33794 | 0.151853 | -1.15409 | 7.62E-05 | 0.000282 |
| ENO1-AS1 | 0.391657 | 0.176468 | -1.15018 | 5.66E-12 | 1.24E-10 |
| LINC00470 | 0.424463 | 0.192394 | -1.14157 | 0.007259 | 0.014486 |
| AC131391.1 | 0.21551 | 0.09782 | -1.13956 | 0.001661 | 0.004047 |
| AL136162.1 | 0.501061 | 0.227887 | -1.13667 | 9.74E-19 | 7.30E-17 |
| BOK-AS1 | 0.817045 | 0.372172 | -1.13445 | 0.011245 | 0.021081 |
| LINC02404 | 0.948282 | 0.433869 | -1.12806 | 0.004444 | 0.009582 |
| LINC00513 | 0.887399 | 0.40795 | -1.12119 | 0.001359 | 0.0034 |
| AC024941.2 | 0.199846 | 0.091928 | -1.12031 | 1.38E-05 | 6.26E-05 |
| AL590705.1 | 0.853534 | 0.393595 | -1.11674 | 4.35E-07 | 2.89E-06 |
| AC024361.2 | 0.578483 | 0.267604 | -1.11217 | 2.12E-13 | 6.83E-12 |
| AC016723.1 | 0.333776 | 0.155863 | -1.0986 | 5.38E-06 | 2.75E-05 |
| AC106900.2 | 0.765243 | 0.359348 | -1.09054 | 0.025613 | 0.043079 |
| AL109615.3 | 0.997989 | 0.46919 | -1.08885 | 0.001721 | 0.00417 |
| DSCR8 | 1.615643 | 0.762127 | -1.084 | 0.010639 | 0.020061 |
| LINC00348 | 0.699367 | 0.330643 | -1.08077 | 0.018888 | 0.033164 |
| AC110285.2 | 2.088199 | 0.990765 | -1.07565 | 5.92E-08 | 4.84E-07 |
| HCG14 | 0.407902 | 0.193631 | -1.07491 | 5.31E-09 | 5.57E-08 |
| LINC01484 | 0.750545 | 0.356398 | -1.07445 | 8.20E-12 | 1.72E-10 |
| AL355482.1 | 0.486832 | 0.231255 | -1.07394 | 0.020863 | 0.03605 |
| AC005670.1 | 0.220574 | 0.104991 | -1.071 | 7.87E-07 | 4.92E-06 |
| LINC01428 | 0.323418 | 0.15442 | -1.06654 | 1.35E-08 | 1.28E-07 |
| AC005841.1 | 0.151938 | 0.07274 | -1.06266 | 0.003883 | 0.008555 |
| AC091153.3 | 0.573588 | 0.275575 | -1.05757 | 2.06E-09 | 2.46E-08 |
| LINC02335 | 0.403051 | 0.193678 | -1.0573 | 0.001143 | 0.002941 |
| MIR210HG | 1.913983 | 0.924786 | -1.04939 | 1.65E-08 | 1.51E-07 |
| AC021146.12 | 0.711977 | 0.344047 | -1.04923 | 0.001271 | 0.003222 |
| LINC00570 | 0.282802 | 0.136968 | -1.04596 | 0.007826 | 0.015481 |
| LINC01446 | 0.26517 | 0.12901 | -1.03943 | 0.006711 | 0.013564 |
| AC008708.1 | 0.916979 | 0.446672 | -1.03767 | 0.006424 | 0.013034 |
| AC083841.1 | 10.33904 | 5.04171 | -1.03612 | 7.77E-08 | 6.18E-07 |
| LINC02476 | 0.706335 | 0.348614 | -1.01872 | 0.001891 | 0.004537 |
| AL354950.1 | 0.219017 | 0.108126 | -1.01833 | 0.022802 | 0.038828 |
| AC027688.1 | 0.732681 | 0.362252 | -1.01619 | 2.27E-06 | 1.27E-05 |
| AC005534.1 | 0.285252 | 0.141267 | -1.01381 | 1.48E-10 | 2.42E-09 |
| AL359313.1 | 0.162448 | 0.080534 | -1.01231 | 0.016316 | 0.0292 |
| AC112219.2 | 0.295363 | 0.146612 | -1.01049 | 3.86E-08 | 3.26E-07 |
| LINC02127 | 0.372114 | 0.185185 | -1.00678 | 7.07E-07 | 4.49E-06 |
| AC007406.1 | 0.264777 | 0.131848 | -1.00591 | 0.000198 | 0.000647 |
| TPRG1-AS1 | 8.644025 | 4.314096 | -1.00264 | 0.004632 | 0.009935 |
| AC025580.3 | 0.153886 | 0.076826 | -1.0022 | 1.36E-09 | 1.69E-08 |
| LINC02055 | 0.477368 | 0.2385 | -1.00111 | 0.004333 | 0.009388 |
| Z93930.3 | 0.313587 | 0.156783 | -1.0001 | 0.016432 | 0.029374 |
| AC079015.1 | 0.190127 | 0.381614 | 1.005154 | 3.84E-09 | 4.24E-08 |
| AC008759.3 | 0.110304 | 0.22181 | 1.007844 | 3.35E-06 | 1.80E-05 |
| LINC01537 | 0.061974 | 0.124982 | 1.011979 | 1.66E-12 | 4.02E-11 |
| AC004130.1 | 0.169978 | 0.344381 | 1.018653 | 5.52E-08 | 4.55E-07 |
| AC004687.1 | 0.42105 | 0.855859 | 1.023381 | 1.08E-07 | 8.29E-07 |
| AC107294.3 | 0.08536 | 0.175252 | 1.037803 | 1.15E-10 | 1.91E-09 |
| AC090409.1 | 0.087372 | 0.179978 | 1.042582 | 2.46E-11 | 4.51E-10 |
| LINC01871 | 1.153705 | 2.392099 | 1.052003 | 6.03E-12 | 1.30E-10 |
| ITGB2-AS1 | 0.308937 | 0.642639 | 1.056696 | 2.79E-10 | 4.28E-09 |
| LINC00426 | 0.090817 | 0.189545 | 1.061505 | 8.82E-13 | 2.36E-11 |
| AL512274.1 | 0.086468 | 0.180493 | 1.0617 | 0.001481 | 0.003669 |
| AC110995.1 | 0.138122 | 0.291312 | 1.076621 | 9.57E-13 | 2.54E-11 |
| AL049838.1 | 0.115765 | 0.24514 | 1.082408 | 1.38E-13 | 4.60E-12 |
| CASC15 | 0.077433 | 0.164234 | 1.084737 | 3.14E-10 | 4.75E-09 |
| VIM-AS1 | 0.16548 | 0.352981 | 1.092937 | 1.39E-20 | 1.69E-18 |
| LINC01914 | 0.13356 | 0.285546 | 1.096235 | 3.22E-13 | 9.87E-12 |
| AC011899.2 | 0.130123 | 0.278234 | 1.096427 | 2.00E-19 | 1.76E-17 |
| LINC00892 | 0.093376 | 0.200527 | 1.102672 | 5.36E-11 | 9.45E-10 |
| LINC02285 | 0.080968 | 0.17396 | 1.103338 | 4.37E-15 | 2.09E-13 |
| HID1-AS1 | 0.069888 | 0.150218 | 1.10395 | 8.60E-15 | 3.57E-13 |
| AC109446.3 | 0.069683 | 0.150297 | 1.108929 | 2.83E-09 | 3.25E-08 |
| AC015819.1 | 0.161084 | 0.347875 | 1.110753 | 3.63E-16 | 1.98E-14 |
| AC006059.1 | 0.087652 | 0.189808 | 1.114682 | 5.75E-06 | 2.92E-05 |
| AL161935.3 | 0.153134 | 0.332676 | 1.119322 | 7.55E-15 | 3.31E-13 |
| AL135818.1 | 0.077191 | 0.167764 | 1.11992 | 1.00E-13 | 3.44E-12 |
| AC145098.1 | 0.143431 | 0.312945 | 1.125556 | 1.49E-11 | 2.94E-10 |
| AC130371.2 | 0.114284 | 0.249474 | 1.126271 | 1.98E-14 | 7.53E-13 |
| PRKCQ-AS1 | 0.121241 | 0.264991 | 1.128062 | 4.60E-15 | 2.13E-13 |
| AC008050.1 | 0.100118 | 0.219263 | 1.130954 | 7.21E-16 | 3.67E-14 |
| AC008105.3 | 0.183029 | 0.401731 | 1.134161 | 1.21E-13 | 4.07E-12 |
| AC022730.4 | 0.08447 | 0.187155 | 1.147726 | 0.001684 | 0.004092 |
| AL357054.4 | 0.064408 | 0.142802 | 1.148715 | 7.42E-20 | 6.89E-18 |
| AC147067.2 | 0.149787 | 0.332192 | 1.149102 | 2.15E-12 | 5.13E-11 |
| AC010175.1 | 0.276308 | 0.618054 | 1.161458 | 8.08E-06 | 3.93E-05 |
| AC015922.3 | 0.457874 | 1.03019 | 1.169888 | 1.17E-29 | 3.70E-27 |
| LINC01943 | 0.215944 | 0.489861 | 1.181718 | 2.08E-20 | 2.27E-18 |
| AL583785.1 | 0.072569 | 0.165117 | 1.18607 | 1.70E-13 | 5.58E-12 |
| AC144831.1 | 0.087463 | 0.199084 | 1.186635 | 2.59E-14 | 9.28E-13 |
| LINC01614 | 0.142629 | 0.324666 | 1.18669 | 7.15E-10 | 9.69E-09 |
| AC129507.1 | 0.068635 | 0.156334 | 1.187611 | 7.86E-13 | 2.12E-11 |
| MIR155HG | 0.22149 | 0.507356 | 1.195755 | 4.63E-13 | 1.34E-11 |
| ACTA2-AS1 | 0.110285 | 0.255595 | 1.212623 | 9.00E-11 | 1.54E-09 |
| AL078590.3 | 0.13173 | 0.306405 | 1.217857 | 6.68E-17 | 4.05E-15 |
| HOTAIRM1 | 0.399233 | 0.930927 | 1.221437 | 4.07E-20 | 4.14E-18 |
| HLA-DQB1-AS1 | 0.4085 | 0.955131 | 1.225363 | 1.10E-15 | 5.45E-14 |
| AC012645.3 | 0.08446 | 0.19821 | 1.230695 | 3.10E-11 | 5.62E-10 |
| LINC02084 | 0.159987 | 0.378349 | 1.241762 | 6.03E-11 | 1.06E-09 |
| AL596442.2 | 0.160363 | 0.379475 | 1.242665 | 4.43E-13 | 1.29E-11 |
| AC009041.2 | 0.083459 | 0.198652 | 1.251108 | 4.04E-25 | 9.11E-23 |
| AGAP2-AS1 | 0.794369 | 1.915306 | 1.269694 | 3.23E-31 | 1.46E-28 |
| HOXB-AS1 | 0.173222 | 0.417727 | 1.269938 | 8.48E-27 | 2.23E-24 |
| LINC00920 | 0.117747 | 0.284843 | 1.274481 | 2.05E-22 | 2.82E-20 |
| AC243960.1 | 0.221006 | 0.535597 | 1.27706 | 3.81E-16 | 2.02E-14 |
| AP000812.1 | 0.056093 | 0.138371 | 1.302653 | 1.38E-14 | 5.53E-13 |
| AC025031.1 | 0.069825 | 0.172761 | 1.306966 | 6.53E-12 | 1.40E-10 |
| LINC02593 | 0.085456 | 0.21195 | 1.310468 | 1.22E-12 | 3.08E-11 |
| TRG-AS1 | 0.108318 | 0.27008 | 1.318115 | 1.82E-19 | 1.64E-17 |
| AL133371.2 | 0.21921 | 0.548989 | 1.324466 | 1.36E-16 | 7.79E-15 |
| LINC01150 | 0.116521 | 0.292617 | 1.32842 | 8.06E-15 | 3.44E-13 |
| AP002761.3 | 0.060567 | 0.152663 | 1.333734 | 7.72E-10 | 1.03E-08 |
| WDR86-AS1 | 0.110489 | 0.279486 | 1.338873 | 1.07E-15 | 5.37E-14 |
| PCED1B-AS1 | 0.580485 | 1.47391 | 1.344318 | 2.79E-21 | 3.52E-19 |
| AC004847.1 | 0.09683 | 0.246163 | 1.34609 | 7.35E-13 | 2.00E-11 |
| AC015922.2 | 0.308357 | 0.785558 | 1.349117 | 1.07E-16 | 6.26E-15 |
| LINC00996 | 0.084395 | 0.217376 | 1.36497 | 4.49E-15 | 2.11E-13 |
| AC018755.4 | 0.220074 | 0.573539 | 1.381906 | 5.28E-10 | 7.35E-09 |
| AC104083.1 | 0.983462 | 2.563841 | 1.382365 | 1.70E-30 | 6.69E-28 |
| AF127936.1 | 0.057924 | 0.151175 | 1.383987 | 5.45E-15 | 2.49E-13 |
| AC026369.3 | 0.070073 | 0.184164 | 1.394063 | 4.36E-10 | 6.29E-09 |
| LOXL1-AS1 | 0.115629 | 0.304941 | 1.399025 | 1.72E-20 | 2.01E-18 |
| HSPC324 | 0.270743 | 0.722992 | 1.417054 | 6.97E-23 | 1.05E-20 |
| AF131215.5 | 0.098076 | 0.265372 | 1.436049 | 4.39E-17 | 2.77E-15 |
| AC245128.3 | 0.066714 | 0.180562 | 1.43644 | 2.24E-08 | 1.97E-07 |
| AL034397.3 | 0.119377 | 0.325342 | 1.446437 | 4.44E-18 | 2.98E-16 |
| AC244153.1 | 0.1096 | 0.303048 | 1.467302 | 5.90E-28 | 1.69E-25 |
| SMIM25 | 0.33678 | 0.93718 | 1.476521 | 1.62E-11 | 3.18E-10 |
| LINC01094 | 0.091564 | 0.256347 | 1.48524 | 4.10E-16 | 2.12E-14 |
| AC015911.3 | 0.085673 | 0.239884 | 1.485433 | 1.40E-14 | 5.53E-13 |
| AC004585.1 | 0.170798 | 0.478885 | 1.487391 | 1.22E-14 | 4.93E-13 |
| TRBV11-2 | 0.109439 | 0.308159 | 1.49354 | 6.22E-08 | 5.06E-07 |
| AL590648.3 | 0.078127 | 0.222142 | 1.507595 | 3.59E-19 | 2.91E-17 |
| AP005019.1 | 0.073111 | 0.213236 | 1.544289 | 6.51E-20 | 6.39E-18 |
| AC090559.1 | 0.178186 | 0.524321 | 1.557069 | 6.84E-19 | 5.40E-17 |
| LINC02446 | 0.140534 | 0.417485 | 1.570802 | 2.26E-11 | 4.17E-10 |
| BX322234.1 | 0.080014 | 0.238074 | 1.573092 | 1.05E-22 | 1.50E-20 |
| LINC02104 | 0.089022 | 0.264946 | 1.573465 | 3.64E-16 | 1.98E-14 |
| AC002091.1 | 0.070223 | 0.210281 | 1.582302 | 4.95E-18 | 3.26E-16 |
| AC013264.1 | 0.068015 | 0.203733 | 1.582759 | 1.59E-12 | 3.88E-11 |
| ROCR | 0.099436 | 0.29822 | 1.584536 | 0.007959 | 0.015715 |
| LINC01197 | 0.068827 | 0.206727 | 1.586679 | 2.16E-38 | 6.83E-35 |
| LINC00861 | 0.098283 | 0.296992 | 1.595408 | 3.45E-14 | 1.22E-12 |
| AP001189.1 | 0.058914 | 0.179605 | 1.608153 | 3.26E-16 | 1.84E-14 |
| AP007216.2 | 0.085917 | 0.263591 | 1.617291 | 3.79E-12 | 8.67E-11 |
| AL136084.3 | 0.110026 | 0.339182 | 1.624214 | 4.72E-17 | 2.92E-15 |
| AP000757.1 | 0.13646 | 0.42116 | 1.625896 | 6.69E-08 | 5.41E-07 |
| AP002954.1 | 0.088624 | 0.276566 | 1.641857 | 1.04E-12 | 2.69E-11 |
| LINC02331 | 0.259374 | 0.813094 | 1.64839 | 0.000382 | 0.001158 |
| AC245041.2 | 0.238286 | 0.761788 | 1.676697 | 0.00017 | 0.000564 |
| HCG11 | 0.097153 | 0.311619 | 1.681457 | 4.39E-36 | 3.47E-33 |
| AC115522.1 | 0.051718 | 0.169623 | 1.713586 | 9.97E-19 | 7.30E-17 |
| NKILA | 0.076916 | 0.254556 | 1.726631 | 2.87E-24 | 4.76E-22 |
| LINC01679 | 0.07181 | 0.240147 | 1.741655 | 1.50E-37 | 2.36E-34 |
| AC027031.2 | 0.205353 | 0.702035 | 1.773436 | 4.78E-12 | 1.08E-10 |
| LINC00924 | 0.195242 | 0.667949 | 1.774472 | 4.19E-32 | 2.21E-29 |
| AL365361.1 | 0.103088 | 0.364074 | 1.820351 | 2.55E-25 | 6.19E-23 |
| LINC02273 | 0.051877 | 0.183766 | 1.824704 | 4.29E-18 | 2.95E-16 |
| MIAT | 0.076338 | 0.274134 | 1.844409 | 1.20E-10 | 1.99E-09 |
| AC010457.1 | 0.095728 | 0.348547 | 1.864342 | 0.009281 | 0.017922 |
| LINC01857 | 0.222376 | 0.845814 | 1.927342 | 3.10E-20 | 3.27E-18 |
| AC109479.1 | 0.061303 | 0.240686 | 1.973124 | 1.02E-18 | 7.30E-17 |
| AC018529.1 | 0.055846 | 0.22642 | 2.019476 | 7.48E-19 | 5.76E-17 |
| DNM3OS | 0.068092 | 0.276577 | 2.022131 | 7.63E-33 | 4.82E-30 |
| LINC01480 | 0.340647 | 1.42175 | 2.06132 | 2.19E-12 | 5.19E-11 |
| AP001189.3 | 0.14329 | 0.598704 | 2.062903 | 7.23E-22 | 9.51E-20 |
| LINC01615 | 0.063966 | 0.279151 | 2.125678 | 3.66E-15 | 1.78E-13 |
| AC100803.2 | 0.104753 | 0.484931 | 2.210791 | 9.03E-30 | 3.17E-27 |
| AC010547.2 | 0.391943 | 1.872006 | 2.25587 | 3.10E-12 | 7.29E-11 |
| AC002398.2 | 0.076075 | 0.375955 | 2.305063 | 1.66E-07 | 1.21E-06 |
| UCA1 | 0.723852 | 3.909673 | 2.433281 | 2.40E-05 | 0.000101 |
| AL049629.1 | 0.103137 | 0.57635 | 2.482379 | 8.78E-08 | 6.91E-07 |
| LINC01133 | 0.039585 | 0.255419 | 2.689834 | 9.51E-11 | 1.61E-09 |
| LINC02544 | 0.048309 | 0.359805 | 2.896837 | 1.88E-24 | 3.48E-22 |
| HAND2-AS1 | 0.018681 | 0.184959 | 3.307587 | 3.59E-36 | 3.47E-33 |

FDR: false discovery rate;
