# Supplementary material for: Identification of Novel Tumor Microenvironment-Related Long Noncoding RNAs to Determine the Prognosis and Response to Immunotherapy of Hepatocellular Carcinoma Patients
Source: Front Mol Biosci. 2021 Dec 24;8:781307. doi: 10.3389/fmolb.2021.781307 (PMC8739902; doi:10.3389/fmolb.2021.781307)
Supplement: Supplementary file 3 [file Table3.DOCX]

Table S3. Differential expression lncRNAs were identified in the immune scores group

| **Gene** | **Low immune scores group** | **High immune scores group** | **logFC** | **pValue** | **FDR** |
| --- | --- | --- | --- | --- | --- |
| AP000593.3 | 2.580447 | 0.412863 | -2.64388 | 0.011322 | 0.025862 |
| LINC02587 | 0.488613 | 0.113418 | -2.10704 | 8.78E-05 | 0.000487 |
| AL590483.2 | 0.388899 | 0.091194 | -2.09239 | 8.94E-06 | 7.07E-05 |
| AC007277.1 | 0.205009 | 0.055336 | -1.8894 | 6.60E-06 | 5.39E-05 |
| AC069294.1 | 0.571551 | 0.183748 | -1.63715 | 4.11E-06 | 3.63E-05 |
| GDNF-AS1 | 0.279848 | 0.092583 | -1.59582 | 0.013048 | 0.028959 |
| AC026765.2 | 1.257699 | 0.43437 | -1.53379 | 7.25E-05 | 0.000414 |
| AC011747.1 | 0.282985 | 0.102527 | -1.46472 | 5.81E-06 | 4.87E-05 |
| BX470209.1 | 0.228774 | 0.084154 | -1.44281 | 0.002043 | 0.006411 |
| AC104088.1 | 0.269908 | 0.09987 | -1.43435 | 4.78E-05 | 0.000295 |
| AC005841.1 | 0.162699 | 0.061979 | -1.39236 | 0.000117 | 0.000615 |
| AL163953.1 | 0.562275 | 0.222884 | -1.33498 | 5.60E-06 | 4.71E-05 |
| LINC02029 | 0.647921 | 0.2582 | -1.32733 | 0.009594 | 0.022503 |
| LINC01970 | 0.180756 | 0.074063 | -1.28721 | 0.000127 | 0.00066 |
| MIR325HG | 0.350207 | 0.14481 | -1.27405 | 0.003672 | 0.01048 |
| AL133153.2 | 0.160616 | 0.066673 | -1.26845 | 0.000437 | 0.001816 |
| BX470209.2 | 0.276504 | 0.115533 | -1.25899 | 0.001811 | 0.00584 |
| AC010501.1 | 0.255631 | 0.10695 | -1.25713 | 1.60E-08 | 2.91E-07 |
| AC006037.1 | 0.465485 | 0.197108 | -1.23975 | 1.11E-05 | 8.55E-05 |
| LINC01549 | 2.989365 | 1.267822 | -1.23749 | 0.001405 | 0.004762 |
| AC008549.1 | 15.87124 | 6.754878 | -1.23241 | 0.000105 | 0.000562 |
| LINC00864 | 0.744474 | 0.320612 | -1.21539 | 0.003937 | 0.011123 |
| LINC01124 | 4.768264 | 2.060553 | -1.21043 | 1.69E-05 | 0.000123 |
| AC010531.5 | 0.522772 | 0.226595 | -1.20607 | 0.000542 | 0.002169 |
| AC113404.1 | 0.515642 | 0.228246 | -1.17578 | 0.000241 | 0.001115 |
| AC004593.1 | 0.47896 | 0.213644 | -1.1647 | 0.000701 | 0.002669 |
| C5orf66 | 0.218697 | 0.098357 | -1.15284 | 0.0002 | 0.000952 |
| AC060780.1 | 1.1147 | 0.504939 | -1.14247 | 6.49E-08 | 9.76E-07 |
| AC007406.1 | 0.272938 | 0.123686 | -1.14189 | 3.72E-07 | 4.52E-06 |
| RNF157-AS1 | 0.483046 | 0.222743 | -1.11678 | 0.004093 | 0.011521 |
| AC007406.2 | 1.623707 | 0.75619 | -1.10247 | 0.018028 | 0.037415 |
| Z82246.1 | 1.704281 | 0.804846 | -1.08238 | 0.007627 | 0.018753 |
| AC092802.3 | 0.195797 | 0.092601 | -1.08025 | 0.002023 | 0.006374 |
| AC090150.1 | 4.01682 | 1.900549 | -1.07964 | 0.000837 | 0.003074 |
| AL023583.1 | 0.243029 | 0.115131 | -1.07785 | 1.45E-05 | 0.000107 |
| LINC00886 | 1.560157 | 0.741582 | -1.07301 | 5.91E-06 | 4.93E-05 |
| LINC00844 | 28.82052 | 13.74089 | -1.06862 | 0.020049 | 0.040848 |
| GCC2-AS1 | 0.404429 | 0.193847 | -1.06097 | 1.33E-08 | 2.47E-07 |
| AC011005.4 | 0.544259 | 0.262053 | -1.05444 | 1.24E-10 | 3.06E-09 |
| AC231981.1 | 0.571266 | 0.275286 | -1.05323 | 3.45E-07 | 4.27E-06 |
| LINC01535 | 0.253657 | 0.12251 | -1.04998 | 0.000441 | 0.001826 |
| AL139161.1 | 1.954854 | 0.95777 | -1.02931 | 0.001068 | 0.003754 |
| AC004862.1 | 2.253968 | 1.111594 | -1.01984 | 0.002304 | 0.007105 |
| AL021068.1 | 0.671524 | 0.332466 | -1.01423 | 3.58E-06 | 3.25E-05 |
| SLC6A1-AS1 | 0.741064 | 0.367866 | -1.01042 | 1.87E-06 | 1.84E-05 |
| ELOVL2-AS1 | 0.621902 | 0.309687 | -1.00588 | 9.54E-08 | 1.35E-06 |
| AL360181.1 | 3.689454 | 1.840502 | -1.00331 | 4.80E-08 | 7.52E-07 |
| AC025031.1 | 0.080773 | 0.161813 | 1.002384 | 5.66E-05 | 0.000339 |
| AC083837.1 | 0.091443 | 0.183405 | 1.004088 | 8.37E-08 | 1.21E-06 |
| AL645939.4 | 0.190829 | 0.385802 | 1.015579 | 0.009283 | 0.021927 |
| AL583785.1 | 0.078542 | 0.159143 | 1.018777 | 5.65E-08 | 8.63E-07 |
| LINC01503 | 0.943591 | 1.919466 | 1.024472 | 4.84E-11 | 1.32E-09 |
| AL078590.3 | 0.144241 | 0.293893 | 1.026805 | 2.41E-09 | 5.08E-08 |
| AL451069.1 | 0.137871 | 0.28164 | 1.030533 | 0.000333 | 0.00147 |
| LOXL1-AS1 | 0.137952 | 0.282618 | 1.034685 | 1.04E-12 | 3.27E-11 |
| AL355512.1 | 1.385122 | 2.863067 | 1.047548 | 0.000359 | 0.001557 |
| MIR3945HG | 0.061644 | 0.127471 | 1.048149 | 6.52E-08 | 9.76E-07 |
| AL158151.4 | 0.07167 | 0.148979 | 1.055662 | 0.008684 | 0.020801 |
| U62317.1 | 0.518625 | 1.082911 | 1.06215 | 2.03E-16 | 8.74E-15 |
| AC092809.2 | 0.098595 | 0.205976 | 1.062896 | 1.02E-09 | 2.22E-08 |
| AL357054.4 | 0.066118 | 0.141092 | 1.093518 | 1.51E-13 | 5.25E-12 |
| FAM83A-AS1 | 4.343283 | 9.28004 | 1.095345 | 4.88E-05 | 0.0003 |
| AC147651.3 | 0.537191 | 1.148364 | 1.096073 | 2.19E-25 | 2.48E-23 |
| LINC01480 | 0.560948 | 1.201449 | 1.098835 | 3.58E-23 | 2.80E-21 |
| AC100803.2 | 0.187586 | 0.402098 | 1.099992 | 2.03E-06 | 1.97E-05 |
| AL133370.1 | 0.255389 | 0.54812 | 1.101796 | 0.007433 | 0.01841 |
| MSC-AS1 | 0.388821 | 0.849051 | 1.126746 | 2.79E-12 | 8.43E-11 |
| HCG11 | 0.128316 | 0.280456 | 1.128075 | 2.12E-14 | 8.08E-13 |
| AL390729.1 | 0.122462 | 0.267929 | 1.129514 | 5.48E-17 | 2.42E-15 |
| LINC00607 | 0.146457 | 0.320728 | 1.130875 | 0.000111 | 0.000589 |
| TNRC6C-AS1 | 0.372119 | 0.823786 | 1.146505 | 2.89E-23 | 2.33E-21 |
| AC096751.2 | 0.112429 | 0.24952 | 1.150144 | 4.39E-07 | 5.23E-06 |
| DNM3OS | 0.106379 | 0.23829 | 1.163498 | 4.84E-09 | 9.59E-08 |
| AL078604.2 | 0.129032 | 0.289074 | 1.163709 | 1.69E-07 | 2.22E-06 |
| AC115522.1 | 0.067965 | 0.153375 | 1.174197 | 4.43E-10 | 1.02E-08 |
| WDR86-AS1 | 0.118698 | 0.271277 | 1.192477 | 3.01E-12 | 9.01E-11 |
| PRKCQ-AS1 | 0.116893 | 0.269339 | 1.204241 | 7.65E-25 | 8.34E-23 |
| LINC02544 | 0.123276 | 0.284838 | 1.208252 | 1.81E-05 | 0.000129 |
| AC011294.1 | 0.226663 | 0.528715 | 1.22194 | 3.83E-06 | 3.42E-05 |
| AC007639.1 | 0.302265 | 0.707499 | 1.226912 | 0.00081 | 0.002994 |
| AC008759.3 | 0.099165 | 0.232948 | 1.232103 | 5.49E-08 | 8.43E-07 |
| AC145098.1 | 0.135055 | 0.321321 | 1.250473 | 2.99E-10 | 7.03E-09 |
| AC021188.1 | 0.070397 | 0.167851 | 1.253599 | 1.62E-19 | 8.84E-18 |
| LINC01679 | 0.091319 | 0.220638 | 1.272699 | 1.36E-18 | 6.83E-17 |
| LINC01914 | 0.12211 | 0.296995 | 1.282257 | 2.39E-10 | 5.71E-09 |
| AP000812.1 | 0.056626 | 0.137838 | 1.283433 | 2.97E-13 | 1.02E-11 |
| AC138207.5 | 0.962427 | 2.35185 | 1.289048 | 8.04E-29 | 1.44E-26 |
| AC007220.1 | 0.164213 | 0.402629 | 1.293883 | 3.50E-07 | 4.31E-06 |
| AL162424.1 | 0.061008 | 0.150767 | 1.305258 | 4.94E-05 | 0.000303 |
| AC004865.2 | 0.094699 | 0.236468 | 1.32023 | 4.98E-20 | 2.87E-18 |
| Z84484.1 | 0.065009 | 0.165637 | 1.349307 | 1.55E-10 | 3.77E-09 |
| AL161935.3 | 0.136715 | 0.349096 | 1.352456 | 7.91E-18 | 3.60E-16 |
| AL590648.3 | 0.084392 | 0.215877 | 1.355023 | 1.50E-14 | 5.97E-13 |
| AC018529.1 | 0.078638 | 0.203628 | 1.372631 | 6.60E-09 | 1.28E-07 |
| MMP2-AS1 | 0.065691 | 0.172169 | 1.390052 | 2.77E-11 | 7.77E-10 |
| AC022730.4 | 0.074879 | 0.196745 | 1.393699 | 0.005569 | 0.014637 |
| TMEM92-AS1 | 0.256837 | 0.687954 | 1.421458 | 1.58E-11 | 4.50E-10 |
| LINC01150 | 0.110643 | 0.298495 | 1.431792 | 2.15E-14 | 8.10E-13 |
| AC079209.1 | 0.247709 | 0.67129 | 1.438289 | 9.31E-12 | 2.68E-10 |
| LINC01697 | 0.075694 | 0.20632 | 1.446624 | 0.007291 | 0.018222 |
| AC110995.1 | 0.115061 | 0.314373 | 1.450078 | 4.95E-21 | 3.15E-19 |
| AC004921.1 | 0.140474 | 0.38516 | 1.455154 | 7.33E-27 | 1.12E-24 |
| AC011899.2 | 0.108449 | 0.299908 | 1.4675 | 1.13E-24 | 1.15E-22 |
| AC022182.2 | 0.071048 | 0.196765 | 1.469599 | 9.24E-20 | 5.13E-18 |
| AC020658.5 | 0.08084 | 0.225384 | 1.479239 | 2.75E-19 | 1.45E-17 |
| AC006059.1 | 0.073217 | 0.204244 | 1.480049 | 0.001061 | 0.003737 |
| LINC01010 | 0.139412 | 0.392397 | 1.49296 | 6.28E-08 | 9.50E-07 |
| FO393401.1 | 0.085087 | 0.241247 | 1.503498 | 1.38E-22 | 1.03E-20 |
| AC015819.1 | 0.132675 | 0.376284 | 1.503926 | 4.69E-29 | 8.95E-27 |
| LINC00426 | 0.073043 | 0.207319 | 1.505033 | 3.47E-27 | 5.90E-25 |
| LINC01943 | 0.182133 | 0.523672 | 1.523668 | 1.07E-24 | 1.13E-22 |
| AC090559.1 | 0.181222 | 0.521285 | 1.524316 | 6.14E-14 | 2.23E-12 |
| AC011511.5 | 0.059997 | 0.175823 | 1.551147 | 2.46E-20 | 1.47E-18 |
| AL133371.2 | 0.194619 | 0.573579 | 1.559337 | 1.48E-21 | 9.64E-20 |
| LINC01587 | 0.188713 | 0.556433 | 1.560013 | 0.013593 | 0.029928 |
| AC079015.1 | 0.142918 | 0.428823 | 1.585198 | 3.18E-24 | 3.13E-22 |
| LINC01094 | 0.086777 | 0.261134 | 1.589407 | 1.47E-18 | 7.25E-17 |
| LINC02285 | 0.063 | 0.191928 | 1.607152 | 2.48E-26 | 3.03E-24 |
| AC008105.3 | 0.14448 | 0.44028 | 1.607557 | 1.59E-29 | 3.47E-27 |
| LINC00996 | 0.074003 | 0.227767 | 1.621899 | 4.20E-22 | 2.91E-20 |
| AC109479.1 | 0.074013 | 0.227976 | 1.623039 | 3.16E-10 | 7.37E-09 |
| AC027031.2 | 0.22144 | 0.685949 | 1.631187 | 3.97E-07 | 4.79E-06 |
| AL034397.3 | 0.106853 | 0.337865 | 1.660817 | 1.09E-23 | 9.81E-22 |
| AP002954.1 | 0.087274 | 0.277916 | 1.671025 | 1.03E-10 | 2.57E-09 |
| AC002091.1 | 0.064916 | 0.215588 | 1.731642 | 1.04E-18 | 5.32E-17 |
| AC012645.3 | 0.065178 | 0.217492 | 1.738498 | 1.69E-26 | 2.24E-24 |
| AF127936.1 | 0.047942 | 0.161157 | 1.749123 | 2.55E-15 | 1.05E-13 |
| U62317.4 | 0.109242 | 0.374821 | 1.778675 | 3.61E-24 | 3.44E-22 |
| LINC01615 | 0.07448 | 0.268637 | 1.850733 | 5.81E-07 | 6.68E-06 |
| AC098613.1 | 0.125261 | 0.458272 | 1.87127 | 5.67E-20 | 3.21E-18 |
| LINC00892 | 0.062099 | 0.231805 | 1.900264 | 1.73E-34 | 7.56E-32 |
| AL135818.1 | 0.051605 | 0.193351 | 1.905651 | 1.72E-29 | 3.51E-27 |
| AP005019.1 | 0.060105 | 0.226242 | 1.912307 | 1.11E-26 | 1.62E-24 |
| TRG-AS1 | 0.079266 | 0.299131 | 1.915998 | 1.73E-34 | 7.56E-32 |
| AC010457.1 | 0.092729 | 0.351545 | 1.922615 | 0.008964 | 0.021337 |
| AC109446.3 | 0.045891 | 0.17409 | 1.923555 | 1.32E-23 | 1.12E-21 |
| AC011899.3 | 0.051136 | 0.194259 | 1.925575 | 3.53E-19 | 1.83E-17 |
| SMIM25 | 0.263216 | 1.010744 | 1.9411 | 4.43E-22 | 3.00E-20 |
| AC004847.1 | 0.070177 | 0.272816 | 1.958863 | 8.36E-31 | 2.32E-28 |
| AC245128.3 | 0.050321 | 0.196954 | 1.968613 | 3.03E-18 | 1.43E-16 |
| HAND2-AS1 | 0.041102 | 0.162537 | 1.983472 | 2.18E-08 | 3.85E-07 |
| AC015911.3 | 0.063508 | 0.262049 | 2.044819 | 6.44E-22 | 4.27E-20 |
| AC002398.2 | 0.088143 | 0.363888 | 2.045583 | 0.002557 | 0.007731 |
| AC018755.4 | 0.153953 | 0.63966 | 2.054818 | 1.85E-18 | 8.81E-17 |
| HLA-DQB1-AS1 | 0.264301 | 1.09933 | 2.056373 | 1.40E-25 | 1.65E-23 |
| ITGB2-AS1 | 0.181167 | 0.77041 | 2.088307 | 8.22E-36 | 6.28E-33 |
| AC243960.1 | 0.143031 | 0.613573 | 2.10091 | 5.02E-34 | 1.92E-31 |
| AC130456.3 | 0.068068 | 0.292427 | 2.103027 | 2.75E-06 | 2.58E-05 |
| AL365361.1 | 0.086803 | 0.38036 | 2.131554 | 1.47E-22 | 1.07E-20 |
| LINC00861 | 0.073055 | 0.322221 | 2.141003 | 1.24E-30 | 3.15E-28 |
| AC004687.1 | 0.234609 | 1.0423 | 2.151437 | 2.37E-26 | 3.02E-24 |
| LINC02195 | 0.082452 | 0.371255 | 2.170786 | 3.64E-22 | 2.59E-20 |
| LINC02273 | 0.042692 | 0.19295 | 2.176185 | 6.36E-27 | 1.02E-24 |
| AL590764.1 | 0.141246 | 0.653302 | 2.209539 | 3.38E-39 | 5.16E-36 |
| LINC01871 | 0.628085 | 2.917718 | 2.215808 | 9.71E-35 | 5.93E-32 |
| AC026369.3 | 0.04473 | 0.209507 | 2.227681 | 5.89E-14 | 2.17E-12 |
| MIR155HG | 0.126275 | 0.602571 | 2.25456 | 6.85E-34 | 2.32E-31 |
| AC013264.1 | 0.04695 | 0.224798 | 2.259443 | 1.41E-26 | 1.96E-24 |
| PCED1B-AS1 | 0.351927 | 1.702468 | 2.274279 | 4.33E-51 | 1.32E-47 |
| AL391056.1 | 0.224164 | 1.119102 | 2.319712 | 4.60E-06 | 3.99E-05 |
| LINC02084 | 0.089088 | 0.449249 | 2.334217 | 1.43E-32 | 4.37E-30 |
| LINC01857 | 0.167459 | 0.900731 | 2.427292 | 8.01E-30 | 1.88E-27 |
| LINC01133 | 0.039389 | 0.255615 | 2.698098 | 3.32E-10 | 7.68E-09 |
| AC004585.1 | 0.08674 | 0.562942 | 2.698209 | 4.25E-36 | 4.33E-33 |
| TRBV11-2 | 0.054865 | 0.362733 | 2.724961 | 1.98E-20 | 1.21E-18 |
| MIAT | 0.045183 | 0.305289 | 2.756333 | 2.68E-23 | 2.21E-21 |
| AC105446.1 | 0.150749 | 1.090966 | 2.855389 | 7.98E-16 | 3.39E-14 |
| LINC02446 | 0.062319 | 0.495701 | 2.99173 | 1.31E-23 | 1.12E-21 |
| LINC01819 | 0.010276 | 0.252709 | 4.620165 | 0.019628 | 0.040177 |

FDR: false discovery rate;
