# Supplementary material for: Identification of Novel Tumor Microenvironment-Related Long Noncoding RNAs to Determine the Prognosis and Response to Immunotherapy of Hepatocellular Carcinoma Patients
Source: Front Mol Biosci. 2021 Dec 24;8:781307. doi: 10.3389/fmolb.2021.781307 (PMC8739902; doi:10.3389/fmolb.2021.781307)
Supplement: Supplementary file 4 [file Table4.DOCX]

Table S4. Commonly downregulated and upregulated lncRNA both in the high immune and stromal scores groups

| Gene | logFC |
| --- | --- |
| DNM3OS | 1.592814 |
| AC145098.1 | 1.188015 |
| AC022730.4 | 1.270713 |
| LINC02285 | 1.355245 |
| LINC01857 | 2.177317 |
| LOXL1-AS1 | 1.216855 |
| AC013264.1 | 1.921101 |
| AP005019.1 | 1.728298 |
| AC012645.3 | 1.484597 |
| LINC02544 | 2.052544 |
| AL357054.4 | 1.121117 |
| AC004585.1 | 2.0928 |
| LINC00861 | 1.868206 |
| AC245128.3 | 1.702526 |
| LINC01914 | 1.189246 |
| AL590648.3 | 1.431309 |
| LINC01480 | 1.580077 |
| AF127936.1 | 1.566555 |
| AC015819.1 | 1.307339 |
| LINC01679 | 1.507177 |
| AP000812.1 | 1.293043 |
| AL133371.2 | 1.441901 |
| AL583785.1 | 1.102424 |
| AL365361.1 | 1.975952 |
| LINC01943 | 1.352693 |
| HLA-DQB1-AS1 | 1.640868 |
| HCG11 | 1.404766 |
| AC006059.1 | 1.297365 |
| AL135818.1 | 1.512786 |
| MIR155HG | 1.725158 |
| LINC02446 | 2.281266 |
| ITGB2-AS1 | 1.572501 |
| AC010457.1 | 1.893478 |
| AC243960.1 | 1.688985 |
| AC018755.4 | 1.718362 |
| LINC01150 | 1.380106 |
| TRG-AS1 | 1.617056 |
| HAND2-AS1 | 2.64553 |
| LINC02273 | 2.000444 |
| AC109479.1 | 1.798081 |
| AL161935.3 | 1.235889 |
| LINC00426 | 1.283269 |
| AC109446.3 | 1.516242 |
| AC090559.1 | 1.540693 |
| AL034397.3 | 1.553627 |
| AC025031.1 | 1.154675 |
| LINC02084 | 1.78799 |
| SMIM25 | 1.708811 |
| AC115522.1 | 1.443892 |
| AC015911.3 | 1.765126 |
| AC018529.1 | 1.696053 |
| LINC01094 | 1.537324 |
| LINC01133 | 2.693966 |
| AC004847.1 | 1.652476 |
| AC008105.3 | 1.370859 |
| AC002091.1 | 1.656972 |
| LINC00892 | 1.501468 |
| LINC01871 | 1.633906 |
| AC110995.1 | 1.263349 |
| AC027031.2 | 1.702311 |
| AP002954.1 | 1.656441 |
| PRKCQ-AS1 | 1.166151 |
| AC004687.1 | 1.587409 |
| AC008759.3 | 1.119974 |
| AL078590.3 | 1.122331 |
| AC100803.2 | 1.655392 |
| WDR86-AS1 | 1.265675 |
| AC002398.2 | 2.175323 |
| AC079015.1 | 1.295176 |
| LINC00996 | 1.493434 |
| AC026369.3 | 1.810872 |
| PCED1B-AS1 | 1.809299 |
| LINC01615 | 1.988205 |
| TRBV11-2 | 2.109251 |
| MIAT | 2.300371 |
| AC011899.2 | 1.281964 |
| AC104088.1 | -1.75 |
| AC090150.1 | -1.15498 |
| AL590483.2 | -1.88135 |
| C5orf66 | -1.25871 |
| AC007406.2 | -1.2826 |
| AC026765.2 | -1.68538 |
| AC010501.1 | -1.33218 |
| AC231981.1 | -1.1335 |
| AC007277.1 | -1.89145 |
| LINC01970 | -1.48494 |
| AC007406.1 | -1.0739 |
| AC010531.5 | -1.19432 |
| AC011747.1 | -1.5852 |
| AC008549.1 | -1.30317 |
| AL023583.1 | -1.12478 |
| AC005841.1 | -1.22751 |
| AC113404.1 | -1.32862 |
| AP000593.3 | -2.02394 |
| AC069294.1 | -1.54166 |
| MIR325HG | -1.40529 |
| AL163953.1 | -1.50539 |
| LINC01124 | -1.3415 |
| LINC00886 | -1.11682 |
| LINC02587 | -2.14413 |
